# Comparative efficacy of combination regimens based on interventional therapy and immune checkpoint inhibitors (ICIs) in patients with intermediate- and advanced-stage hepatocellular carcinoma: a systematic review, meta-analysis, and network meta-analysis

**DOI:** 10.1007/s00262-025-04251-5

**Published:** 2026-02-07

**Authors:** Jingting Su, Yuejiao Su, Rongyun Mai, Xing Gao, Shizhou Li, Dandan Zeng, Weijie Cen, Zhenbo Huang, Xiaoqing Li, Haoyu Zeng, Wenbing Li, Can Zeng, Tianzhun Wu, Kaixiang Mo, Jiazhou Ye, Yan Lin, Rong Liang

**Affiliations:** 1 Department of Digestive Oncology, Guangxi Medical University Cancer Hospital, Nanning, 530200 Guangxi China; 2 Department of Hepatobiliary Surgery, Guangxi Medical University Cancer Hospital, Nanning, 530200 Guangxi China

**Keywords:** Hepatocellular carcinoma, Immunotherapy, Transarterial interventional therapy, Meta-analysis, Survival outcomes

## Abstract

**Background:**

Combining interventional therapy with immune checkpoint inhibitors (ICIs) has shown potential benefits in hepatocellular carcinoma (HCC). However, comprehensive evidence on its efficacy and safety remains limited.

**Methods:**

A systematic search of PubMed, Embase, Cochrane Library, and Web of Science was conducted to identify eligible studies for single-arm and Bayesian network meta-analyses (NMA). Progression-free survival (PFS) was the primary endpoint, while overall survival (OS), objective response rate (ORR), and grade ≥ 3 adverse events (AEs) were secondary outcomes (PROSPERO: CRD42024619661).

**Findings:**

This study included 45 studies (n = 4,738), evaluating 14 distinct regimens. In single-arm analysis, transcatheter arterial chemoembolization (TACE) plus tyrosine-kinase inhibitor (TKI) plus tislelizumab [TACE-TKI-Tisle] yielded a pooled median PFS of 11.7 months (95% confidence interval [CI] 8.02–15.37), an ORR of 72% (95% CI 63–80%), and a grade ≥ 3 AE rate of 24% (95% CI 15–34%). NMA showed that TACE-TKI-Tisle and TACE-TKI-Camrelizumab (Camre) achieved significantly longer PFS than TACE-TKI or TACE alone. TACE-TKI-Toripalimab (Tori) showed OS benefits over TACE-TKI-Camre (HR = 0.43; 95% CI 0.20–0.95) and TACE-TKI-Pembrolizumab (Pembro) (HR = 0.32; 95% CI 0.13–0.81). Cumulative ranking via surface under the cumulative ranking curve (SUCRA) indicated that TACE-TKI-ICI achieved the highest efficacy ranking. TACE-TKI-Tisle and TACE-TKI-Tori ranked highest for PFS/ORR, with TACE-TKI-Tori ranking first for OS (SUCRA = 0.981). While TACE-TKI-ICI combinations were generally associated with more grade ≥ 3 AEs, TACE-TKI-Tisle ranked intermediately for safety (SUCRA = 0.426).

**Conclusion:**

TACE-TKI-ICI combinations show promising efficacy in HCC. TACE-TKI-Tisle offers balanced efficacy and safety, while TACE-TKI-Tori provides notable OS benefits, warranting further validation in prospective studies.

**Supplementary Information:**

The online version contains supplementary material available at 10.1007/s00262-025-04251-5.

## Introduction

Hepatocellular carcinoma (HCC) ranks sixth in global cancer incidence and is the third leading cause of cancer-related mortality [1]. Early-stage HCC is usually asymptomatic, which often leads to diagnosis at intermediate or advanced stages and thereby reduces the chances of curative treatment [2]. Currently, the main therapeutic approaches for intermediate- and advanced-stage HCC include transarterial interventional therapies and immune checkpoint inhibitor (ICI)-based regimens. Both modalities, when used alone or in combination, have shown promising outcomes in recent clinical studies.

Transarterial interventional therapy constitutes a cornerstone of treatment for intermediate- and advanced-stage HCC and includes procedures such as transcatheter arterial chemoembolization (TACE), transarterial embolization (TAE), transarterial radioembolization (TARE), and hepatic arterial infusion chemotherapy (HAIC) [3]. These approaches exploit the liver’s dual blood supply: normal hepatic tissue primarily receives blood from the portal vein, whereas HCC lesions are mainly supplied by the hepatic artery. This difference enables selective arterial interventions that can effectively target tumor tissue while sparing normal parenchyma. The efficacy of these treatments has been demonstrated in multiple studies [4, 5]. Meanwhile, the introduction of ICIs has substantially expanded the treatment landscape for advanced HCC. Several pivotal clinical trials have confirmed the efficacy of ICI monotherapy and combination regimens. For example, the CheckMate 9DW trial showed that nivolumab plus ipilimumab as a first-line therapy improved 18-month (34% vs. 18%) and 24-month (28% vs. 12%) progression-free survival (PFS) rates compared with lenvatinib or sorafenib (LEN/SOR). The study also reported a longer PFS2 (median progression-free survival on next-line therapy) for the combination regimen (19.3 vs. 15.4 months; hazard ratio [HR] = 0.70, 95% confidence interval [CI]: 0.58–0.84) [6].

Building on the local control advantage of interventional therapy and the systemic immune activation induced by ICIs, the exploration of combination and multi-drug regimens has naturally emerged as a key research focus. Among dual-combination strategies, TACE plus ICI has been widely investigated and has shown encouraging efficacy and acceptable safety profiles. For instance, the phase II PETAL trial reported a 12-week progression-free survival rate of 93% and a median PFS of 8.95 months (95% CI, 7.30-not reached) for TACE combined with pembrolizumab, with manageable toxicity [7]. Regarding triple-combination approaches, regimens combining TACE with tyrosine-kinase inhibitors (TKIs) or anti-angiogenic antibodies and ICIs [TACE-TKI-ICI/anti-VEGF–ICI] are gaining attention. The phase III EMERALD-1 study (TACE + durvalumab + bevacizumab) demonstrated a significant improvement in PFS compared with TACE alone (HR = 0.77, 95% CI: 0.61–0.98) with a tolerable safety profile [8]. Similarly, the LEAP-012 study showed a significant PFS benefit with TACE + lenvatinib + pembrolizumab compared with TACE alone (HR = 0.66, 95% CI: 0.51–0.84) [9], suggesting a potential new treatment option for intermediate- and advanced-stage HCC.

Despite the growing body of research on combination therapies, several key knowledge gaps remain in the current evidence base. First, there is a lack of systematic evaluation of novel ICIs such as tislelizumab in interventional combination settings, and comparative evidence among different ICIs remains limited. Second, although evidence for TACE-TKI-ICI is increasing, integrated analyses comparing various triple-combination strategies (e.g., different TKI and ICI pairings) are still lacking, and data on HAIC-based triple regimens are scarce. Third, evidence guiding the selection between dual and triple combinations, as well as identifying suitable patient subgroups, is still insufficient, thereby limiting the evidence base for clinical decision-making.

Therefore, this study aims to systematically evaluate the efficacy and safety of combination regimens based on interventional therapy and ICIs in patients with intermediate- and advanced-stage HCC. We further compare the clinical performance of various ICI types and explore the value of emerging triple-combination regimens. By addressing these evidence gaps, this study seeks to provide integrated evidence to inform and refine clinical decision-making for the management of intermediate- and advanced-stage HCC.

## Methods

This systematic review adhered to the Preferred Reporting Items for Systematic Reviews and Meta-Analyses (PRISMA) guidelines [10] (Table S1). To ensure transparency and methodological rigor, the study protocol was prospectively registered with PROSPERO (CRD42024619661).

### Literature search and study selection

Studies were considered eligible if they fulfilled the following inclusion criteria: (1) enrolled patients with a pathological or clinical diagnosis of intermediate or advanced-stage HCC; (2) the interventions included at least one study arm with a combination regimen based on interventional therapy and ICIs; (3) the studies included at least one of the following outcomes of interest: PFS, OS, objective remission rate (ORR), or grade ≥ 3 adverse events (AEs); (4) published phase I, II, or III clinical trials or cohort studies. Exclusion criteria comprised: (1) studies involving patients with secondary HCC; (2) clinical studies involving multiple immunological agents and different interventional techniques (e.g., HAIC for some patients and TACE for others); and (3) incomplete or unavailable data on the target outcome.

We conducted a comprehensive search across PubMed, Embase, Cochrane, and Web of Science from database inception through June 2025. The literature search utilized keywords such as “intermediate and advanced-stage hepatocellular carcinoma,” “interventional therapy,” and “immune checkpoint inhibitors” (complete search strategy available in Table S2). Two authors (JTS and YJS) independently screened titles and abstracts and evaluated full-text articles for eligibility, with discrepancies adjudicated by a third author (WJC).

### Data extraction and risk of bias assessment

The primary outcome was PFS, while secondary outcomes included OS, ORR, and the rate of grade ≥ 3 AEs. Relevant data were independently extracted from the included studies by two authors (JTS and YJS) and recorded in a pre-designed Excel sheet. The collected data included baseline characteristics, sample size, treatment modality and dose, OS, PFS, ORR, the rate of grade ≥ 3 Aes, and study design.

We used three tools to assess the methodological quality and potential biases in the included studies: the Cochrane risk of bias tool for randomized controlled trials, the methodological index for non-randomized studies (MINORS) for non-randomized single-arm clinical trials, and the Newcastle–Ottawa Quality Assessment Scale (NOS) for cohort studies. The assessments were independently conducted by two authors (JTS and YJS). All disagreements were resolved through discussion and consensus with a third author (WJC).

### Data analysis

Statistical analyses were performed in R software (v4.4.1), using the meta package for single-arm meta-analyses (metaprop and metamean functions) and the gemtc and JAGS packages for NMA. For the meta-analysis of survival data, hazard ratios were preferred as the outcome measure. If studies did not report HRs, data were extracted and estimated using methods described by Popat et al. and Jayne et al. [11, 12].

We performed a single-arm meta-analysis to derive pooled estimates of median PFS and OS, and to calculate the pooled rates of objective response, and grade ≥ 3 AEs for each treatment regimen. A network meta-analysis (NMA) was conducted using a Bayesian framework with a random-effects consistency model to estimate relative treatment effects. The analysis employed Markov chain Monte Carlo (MCMC) methods with four independent chains using non-informative priors and normal distributions. The number of simulations was set to 20,000, and the number of iterations was set to 50,000 to obtain the posterior distributions. Trajectory plots and Brooks–Gelman–Rubin diagnostic plots were employed to visually assess the level of model convergence. The effect sizes were expressed in terms of hazard ratios or relative risks, along with their respective 95% confidence intervals. Treatment rankings were determined by the probability of superiority and summarized using the surface under the cumulative ranking (SUCRA) values, ranging from 0 (least effective or least toxic treatment) to 1 (the highest efficacy or toxicity). Consistency for direct and indirect evidence was evaluated through node-splitting test. Study heterogeneity was assessed using the Q test and quantified with the I^2^ statistic.

## Results

From an initial pool of 3917 identified records, title and abstract screening excluded 3813 irrelevant studies. Full-text review of 104 articles yielded 45 eligible studies [8, 9, 13–55]. The final analysis included 2 randomized controlled trials, 28 retrospective cohort studies, and 15 single-arm non-randomized trials (n = 4738) evaluating 14 therapeutic regimens (Fig. S10): HAIC plus TKI plus camrelizumab (HAIC-TKI-Camre), TACE plus bevacizumab plus sintilimab (TACE-Bev-Sinti), TACE plus bevacizumab plus atezolizumab (TACE-Bev-Atez), TACE plus durvalumab (TACE-Durva), TACE plus bevacizumab plus durvalumab (TACE-Bev-Durva), TACE plus camrelizumab (TACE-Camre), TACE plus TKI plus camrelizumab (TACE-TKI-Camre), TACE plus TKI plus pembrolizumab (TACE-TKI-Pembro), TACE plus TKI plus sintilimab (TACE-TKI-Sinti), TACE plus TKI plus tislelizumab (TACE-TKI-Tisle), TACE plus TKI plus toripalimab (TACE-TKI-Tori), HAIC plus TKI plus toripalimab (HAIC-TKI-Tori), HAIC plus TKI plus tislelizumab (HAIC-TKI-Tisle), and TAE plus HAIC plus TKI plus tislelizumab (TAE-HAIC-TKI-Tisle). Most studies enrolled patients with Barcelona Clinic Liver Cancer (BCLC) stage B-C and Child–Pugh class A-B cirrhosis. Comprehensive baseline characteristics are provided in Table S3. All included studies were deemed to have a low to moderate risk of bias, supported by clearly defined criteria, explicit objectives, sufficient follow-up, and comprehensive outcome evaluation (Fig.S1, S2, Table S4).

**Fig. 1:**
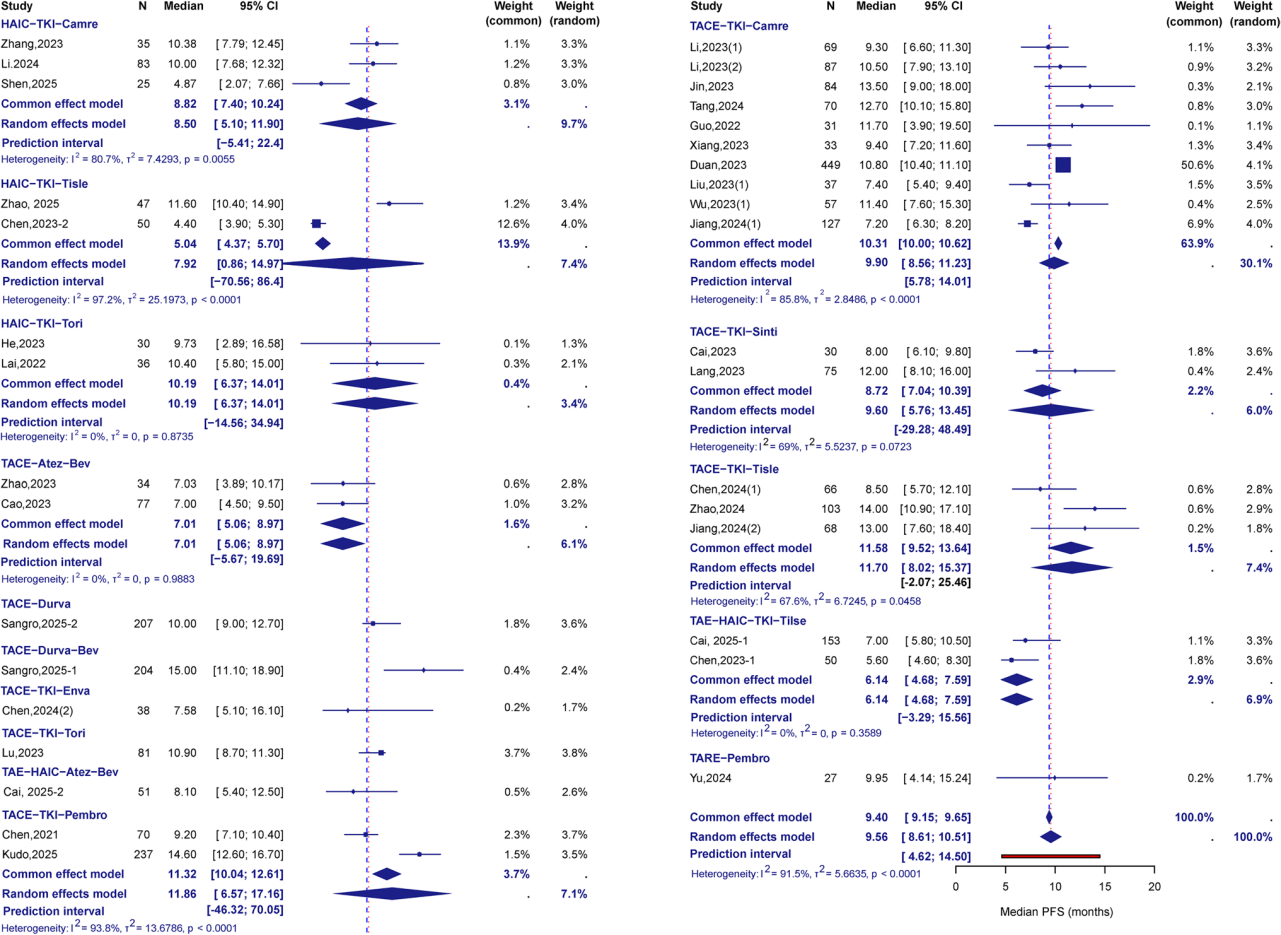
Pooled median Progression-Free Survival (mPFS) of each combined treatment strategies in Single-Arm Meta-Analysis. Forest plot shows the pooled mPFS (and 95% confidence intervals) across treatment regimens, offering a descriptive overview of the strategies. TACE = transcatheter arterial chemoembolization, HAIC = hepatic arterial infusion chemotherapy, TARE = transarterial radioembolization, TKI = tyrosine-kinase inhibitor, Bev = bevacizumab, Camre = camrelizumab, Pembro = pembrolizumab, Tisle = tislelizumab, Tori = toripalimab, Atez = atezolizumab, Sinti = sintilimab, Envafo = envafolimab, Chemo = chemotherapy, Durva = Durvalumab

The single-arm meta-analysis, 31 studies (2921 participants) [8, 9, 16–27, 30–32, 34, 35, 40, 41, 43–47, 49–51, 54, 55] for mPFS, 21 studies (1724 participants) [18, 20–25, 27, 32–35, 40, 41, 44, 47, 49–51, 53, 55] for mOS, 45 studies (3618 participants) [8, 9, 13–55] for ORR, and 24 studies (2384 participants) [8, 9, 13, 14, 16–20, 22–24, 26–30, 32, 34, 35, 39, 43, 48, 54] for grade ≥ 3 AEs rates were included. Among all combination regimens based on interventional therapy and ICIs, the pooled mPFS was 9.56 months (95% CI: 8.61–10.51; I^2^ = 91.5%), the pooled mOS was 18.20 months (95% CI: 16.27–20.13; I^2^ = 96.7%), the pooled ORR was 59% (95% CI: 55–64%; I^2^ = 85.5%), and the pooled incidence of grade ≥ 3 AEs was 37% (95% CI: 29–45%; I^2^ = 91.5%). The descriptive pooled results for specific regimens revealed distinct profiles. For TACE-TKI-Tisle, the pooled mPFS was 11.7 months (95% CI: 8.02–15.37; I^2^ = 67.6%), the pooled ORR was 72% (95% CI: 63–80%; I^2^ = 49.7%), and the pooled incidence of grade ≥ 3 AEs was 24% (95% CI: 15–34%; I^2^ = 65.7%). The pooled ORR for TACE-TKI-Tori was 69% (95% CI: 60–77%; I^2^ = 1.8%). For TACE-TKI-Camre, which had relatively comprehensive data, the pooled mPFS was 9.90 months (95% CI: 8.56–11.23; I^2^ = 85.8%), the pooled mOS was 19.61 months (95% CI: 16.82–22.41; I^2^ = 91.5%), the pooled ORR was 56% (95% CI: 47–64%; I^2^ = 86.4%), and the pooled incidence of grade ≥ 3 AEs was 35% (95% CI: 22–50%; I^2^ = 91.4%). Additionally, for HAIC-TKI-Camre, the pooled incidence of grade ≥ 3 AEs was 78% (95% CI: 71–84%; I^2^ = 0.0%) and the pooled mPFS was 8.50 months (95% CI: 5.10–11.90; I^2^ = 80.7%). These pooled findings from single-arm studies serve as preliminary references for considering the efficacy and safety of different combination strategies (Fig. 1, Fig.S3).

**Fig. 2:**
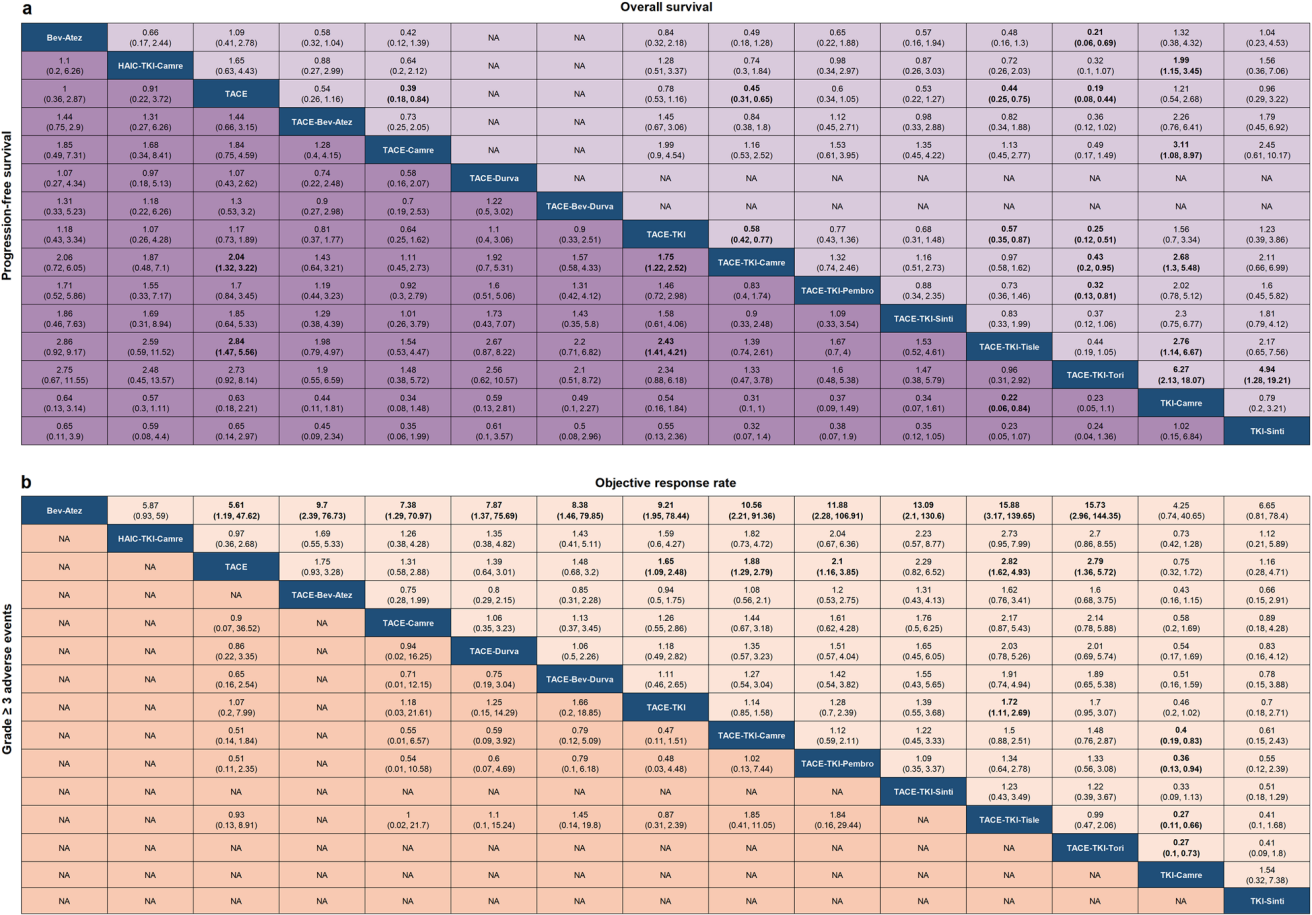
Pooled estimates of the network meta-analysis. **a** Overall survival and Progression-free survival. **b** Objective response rate and grade ≥ 3 adverse events. Data are pooled HR (95% credible interval) for A and RR (95% credible interval) for B. Bold data indicate a significant difference. TACE = transcatheter arterial chemoembolization, HAIC = hepatic arterial infusion chemotherapy, TKI = tyrosine-kinase inhibitor, Bev = bevacizumab, Camre = camrelizumab, Pembro = pembrolizumab, Tisle = tislelizumab, Tori = toripalimab, Atez = atezolizumab, Sinti = sintilimab, Durva = Durvalumab

The network meta-analysis incorporated 27 studies (n = 3,695) [8, 9, 31–55]. Constraints due to insufficient data limited comparisons to 10 interventions for PFS [8, 9, 32–51, 54, 55] and ORR [8, 9, 32–45, 47–55], 8 for OS [9, 32–51, 54, 55], and 6 for grade ≥ 3 AEs incidence [8, 9, 34, 35, 40, 43, 49, 54] (Fig. S11). TACE-TKI-Tisle and TACE-TKI-Camre demonstrated superior progression-free survival compared to TACE-TKI and TACE alone. TACE-TKI-Tori showed overall survival benefits over TACE-TKI-Camre (HR = 0.43; 95% CI 0.20–0.95) and TACE-TKI-Pembro (HR = 0.32; 95% CI 0.13–0.81). In terms of ORR, TACE-TKI-Tisle and TACE-TKI-Tori showed higher effect estimates compared with Bev-Atez, TKI-Camre, and TACE alone. Safety assessment of grade ≥ 3 AEs revealed 5 interventional comparisons without statistically significant differences (Fig. 2).

SUCRA analysis suggested that TACE-TKI-ICI generally obtained higher efficacy rankings than other strategies. For PFS, TACE-TKI-Tisle and TACE-TKI-Tori had the highest SUCRA values (0.896 and 0.831, respectively). Bayesian ranking probability analysis showed that TACE-TKI-Tisle had a 32% probability of being the best regimen and a 31% probability of being the second-best, whereas TACE-TKI-Tori had a 35% probability of being ranked first and a 19% probability of being ranked second. Regarding OS, TACE-TKI-Tori had the highest rank (SUCRA = 0.981) and an 85% probability of being the top-performing regimen. For ORR, both TACE-TKI-Tisle and TACE-TKI-Tori also had high SUCRA values (0.892 and 0.871, respectively). In contrast, regimens combining TKIs with ICIs without interventional therapy (e.g., TKI-Sinti and TKI-Camre) were associated with lower SUCRA values across all efficacy outcomes, with ranking probabilities suggesting they were more likely to be ranked lower. Safety analysis showed a different pattern. TACE-TKI-Pembro (SUCRA = 0.694) and TACE-TKI-Camre (SUCRA = 0.760) had the highest SUCRA values for adverse events, indicating a potentially higher risk of serious toxicity. Notably, TACE-TKI-Tisle, which showed favorable efficacy rankings, was in the middle range for safety (SUCRA = 0.426) (Fig. S12, Table S5).

In the subgroup network meta-analysis, most pairwise comparisons did not reach statistical significance; however, trends favoring the triple combination of TACE-TKI-ICI over dual or monotherapy approaches were observed in several subgroups, including those defined by Child–Pugh A, HBV positivity, ≤ 3 Tumors, prior treatment status, and TACE type. Notably, in treatment-naïve patients, TACE-TKI-Tori was associated with a greater survival benefit (SUCRA = 0.981) than TACE-TKI-Camre (HR = 2.28, 95% CI: 1.14–4.89) and TACE-Bev-Atez (HR = 2.96, 95% CI: 1.22–7.63), suggesting its potential suitability for the first-line setting. The SUCRA ranking analysis offered a comparative perspective on the relative efficacy of different strategies. Among the comparable regimens, TACE-TKI-Tisle and TACE-TKI-Tori tended to rank higher for PFS and OS across most subgroups. However, some subgroup analyses suggested potential variations in treatment rankings. For PFS, TACE-TKI-Sinti ranked higher than TACE-TKI-Tisle in HBV-negative and Child–Pugh B populations, whereas TACE-TKI-Tori was ranked higher than TACE-TKI-Tisle in treatment-naïve patients. For OS, TACE-TKI-Sinti showed a superior ranking to TACE-TKI-Tisle in patients with ECOG 0, while TACE-TKI-Camre demonstrated a better ranking in those with metastatic disease or a history of prior treatment (Fig.S5–S8).

## Discussion

In recent years, treatment strategies for intermediate- and advanced-stage HCC have evolved substantially. The integration of interventional therapies and ICIs has emerged as a major research focus, with multiple pivotal studies confirming the clinical benefits of this combination approach [8, 9]. Collectively, these findings support the concept of comprehensive multimodal treatment for intermediate and advanced-stage HCC and suggest new avenues for optimizing therapeutic strategies in clinical practice.

In this meta-analysis, we performed a multidimensional evaluation of published regimens combining interventional therapies with ICIs. Overall, the TACE-TKI-ICI regimen showed better performance across multiple efficacy endpoints. Among these, TACE-TKI-Tisle showed robust and consistent efficacy data. Meanwhile, TACE-TKI-Tori not only exhibited a stronger OS signal but also achieved high SUCRA rankings for PFS and ORR. The enhanced efficacy of triple-agent regimens likely results from synergistic mechanisms. Previous studies indicate that TACE is associated with lower intra-tumor depletion of effector (CD8+/PD-1+) and regulatory (CD4+FOXP3+) T cells, which may convert the tumor microenvironment from immunosuppressive to immunopermissive, thereby enhancing the response to immunotherapies [56]. TKIs, through their anti-proliferative and anti-angiogenic effects, mitigate the hypoxia-driven neovascularization induced by TACE or HAIC, further limiting HCC progression [57]. Additionally, molecular distinctions among ICIs may also contribute to efficacy differences. Tislelizumab binds the CC′ loop of PD-1, exhibiting slower dissociation and higher affinity, while toripalimab targets the FG loop, blocking classical PD-1 signaling and enhancing memory CD8+ T-cell responses and cytotoxic activity [58, 59]. These structural and functional variations may underlie the distinct efficacy profiles observed across populations and tumor microenvironments, providing a biologically plausible rationale for the outcomes of TACE-TKI-ICI strategies.

While combination therapy offers promising efficacy, its safety profile requires careful consideration. The single-arm meta-analysis revealed a pooled incidence of grade ≥ 3 AEs of 37% (95% CI: 29–45%) for combination regimens based on interventional therapy and ICIs. The network meta-analysis further confirmed, based on SUCRA rankings, that combination regimens generally carry a higher risk of severe AEs. Notably, TACE-TKI-Tisle achieved a favorable balance between efficacy and safety, whereas safety data for TACE-TKI-Tori remain limited. These findings highlight the importance of individualized risk–benefit assessment and toxicity monitoring when selecting combination regimens for clinical use.

Subgroup analyses further revealed potential efficacy heterogeneity. Across most subgroups, TACE-TKI-ICI consistently showed better efficacy; however, efficacy rankings varied in specific populations. For example, in HBV-negative or Child–Pugh B patients, TACE-TKI-Sinti ranked higher than TACE-TKI-Tisle, suggesting that liver function and viral infection status may modulate ICI responsiveness. Previous evidence indicates that HBV infection can reshape the immune microenvironment and influence immunotherapy outcomes [60], while patients with Child–Pugh B cirrhosis experience reduced immunotherapy efficacy and increased mortality risk [61, 62]. These findings underscore the impact of hepatic reserve, viral etiology, and prior treatment on treatment efficacy. These factors not only reflect the impact of patient conditions on immune modulation but may also represent a potential source of heterogeneity in the pooled results. Clinically, this highlights the need for personalized therapy based on functional status and disease stage. Identifying optimal regimens for specific subgroups through stratified analyses will be key to advancing precision medicine in HCC.

This study has several limitations. First, most included studies were retrospective cohort studies or non-randomized single-arm trials. Although approximately half of the retrospective cohorts used propensity score matching (PSM) to reduce intergroup differences, and all single-arm meta-analyses included prospective studies, inherent heterogeneity could not be completely eliminated. Second, for some treatment regimens, the number of eligible studies and sample sizes remained limited. To enhance robustness, we conducted both single-arm and network meta-analyses, which showed generally consistent results. Third, limited data on TKI subtype and hepatitis C virus (HCV) infection precluded further subgroup analysis, and results pertaining to these factors should be interpreted cautiously. Fourth, discrepancies between PFS and OS were observed, which is a common finding in oncology meta-analyses. These differences may be attributable to variations in subsequent therapies (summarized in Table S8), limited OS follow-up, or differences in the mechanisms of action among various ICIs. Incomplete reporting of subsequent treatments and survival outcomes in some studies further warrants cautious interpretation of OS results. Considering these limitations, PFS was adopted as the primary endpoint, as it is less influenced by post-progression therapies and better reflects direct treatment efficacy.

In conclusion, this study provides a comprehensive assessment of the efficacy and safety of combination regimens based on interventional therapy and ICIs in intermediate- and advanced-stage HCC. The TACE-TKI-ICI regimen demonstrated promising efficacy, with TACE-TKI-Tisle showing favorable outcomes across multiple endpoints and an acceptable safety profile. TACE-TKI-Tori exhibited strong OS benefits but had limited safety data. Given the uncertainty in relative efficacy and generalizability, these findings are exploratory but provide useful guidance for future research. Prospective randomized trials are warranted to confirm optimal regimens and define patient populations most likely to benefit.

## Supplementary Information

Below is the link to the electronic supplementary material.

Supplementary file1 (PDF 9100 kb)

Supplementary file2 (DOCX 25026 kb)

## Data Availability

Relevant data supporting this study can be accessed by contacting the corresponding author with a valid rationale.

## References

[1] Kudo M (2010) Management of hepatocellular carcinoma: from prevention to molecular targeted therapy. Oncology 78(Suppl 1):1–6. 10.1159/000315222 20616576 10.1159/000315222

[2] Chidambaranathan-Reghupaty S, Fisher PB, Sarkar D (2021) Hepatocellular carcinoma (HCC): epidemiology, etiology and molecular classification. Adv Cancer Res 149:1–61. 10.1016/bs.acr.2020.10.001 33579421 10.1016/bs.acr.2020.10.001 PMC8796122

[3] Sieghart W, Hucke F, Peck-Radosavljevic M (2015) Transarterial chemoembolization: modalities, indication, and patient selection. J Hepatol 62(5):1187–1195. 10.1016/j.jhep.2015.02.010 25681552 10.1016/j.jhep.2015.02.010

[4] Brown KT, Do RK, Gonen M, Covey AM, Getrajdman GI, Sofocleous CT et al (2016) Randomized trial of hepatic artery embolization for hepatocellular carcinoma using doxorubicin-eluting microspheres compared with embolization with microspheres alone. J Clin Oncol 34(17):2046–2053. 10.1200/JCO.2015.64.0821 26834067 10.1200/JCO.2015.64.0821 PMC4966514

[5] Li QJ, He MK, Chen HW, Fang WQ, Zhou YM, Xu L et al (2022) Hepatic arterial infusion of oxaliplatin, fluorouracil, and leucovorin versus transarterial chemoembolization for large hepatocellular carcinoma: a randomized phase III trial. J Clin Oncol 40(2):150–160. 10.1200/JCO.21.00608 34648352 10.1200/JCO.21.00608

[6] Decaens T, Yau T, Kudo M, Sangro B, Qin S, Da Fonseca L et al (2024) 965MO nivolumab (NIVO) plus ipilimumab (IPI) vs lenvatinib (LEN) or sorafenib (SOR) as first-line (1L) treatment for unresectable hepatocellular carcinoma (uHCC): expanded analyses from CheckMate 9DW. Ann Oncol 35:S657. 10.1016/j.annonc.2024.08.1025. (**S657**)

[7] Pinato DJ, D’Alessio A, Fulgenzi CAM, Schlaak AE, Celsa C, Killmer S et al (2024) Safety and preliminary efficacy of pembrolizumab following transarterial chemoembolization for hepatocellular carcinoma: the PETAL phase Ib study. Clin Cancer Res 30(11):2433–2443. 10.1158/1078-0432.Ccr-24-0177 38578610 10.1158/1078-0432.CCR-24-0177 PMC11145164

[8] Sangro B, Kudo M, Erinjeri JP, Qin S, Ren Z, Chan SL et al (2025) Durvalumab with or without bevacizumab with transarterial chemoembolisation in hepatocellular carcinoma (EMERALD-1): a multiregional, randomised, double-blind, placebo-controlled, phase 3 study. Lancet 405(10474):216–232. 10.1016/S0140-6736(24)02551-0 39798579 10.1016/S0140-6736(24)02551-0 PMC12282661

[9] Kudo M, Ren Z, Guo Y, Han G, Lin H, Zheng J et al (2025) Transarterial chemoembolisation combined with lenvatinib plus pembrolizumab versus dual placebo for unresectable, non-metastatic hepatocellular carcinoma (LEAP-012): a multicentre, randomised, double-blind, phase 3 study. Lancet 405(10474):203–215. 10.1016/S0140-6736(24)02575-3 39798578 10.1016/S0140-6736(24)02575-3

[10] Liberati A, Altman DG, Tetzlaff J, Mulrow C, Gotzsche PC, Ioannidis JP et al (2009) The PRISMA statement for reporting systematic reviews and meta-analyses of studies that evaluate health care interventions: explanation and elaboration. J Clin Epidemiol 62(10):e1–e34. 10.1016/j.jclinepi.2009.06.006 19631507 10.1016/j.jclinepi.2009.06.006

[11] Popat S, Matakidou A, Houlston RS (2004) Thymidylate synthase expression and prognosis in colorectal cancer: a systematic review and meta-analysis. J Clin Oncol 22(3):529–536. 10.1200/JCO.2004.05.064 14752076 10.1200/JCO.2004.05.064

[12] Tierney JF, Stewart LA, Ghersi D, Burdett S, Sydes MR (2007) Practical methods for incorporating summary time-to-event data into meta-analysis. Trials 8:16. 10.1186/1745-6215-8-16 17555582 10.1186/1745-6215-8-16 PMC1920534

[13] Wu X-K, Yang L-F, Chen Y-F, Chen Z-W, Lu H, Shen X-Y et al (2024) Transcatheter arterial chemoembolisation combined with lenvatinib plus camrelizumab as conversion therapy for unresectable hepatocellular carcinoma: a single-arm, multicentre, prospective study. Eclin Med 67:102367. 10.1016/j.eclinm.2023.102367 10.1016/j.eclinm.2023.102367 PMC10758712 38169778

[14] Mu M-Y, Chen Z-X, Cao Y-Z, Fu X-B, Qiu L-J, Qi H et al (2025) Transarterial chemoembolization combined with intra-arterial infusion of sintilimab and bevacizumab for advanced hepatocellular carcinoma: a phase 2 study. Cancer Lett 628:217851. 10.1016/j.canlet.2025.217851 40472923 10.1016/j.canlet.2025.217851

[15] Liu D, Mu H, Liu C, Zhang W, Cui Y, Wu Q et al (2023) Sintilimab, bevacizumab biosimilar, and HAIC for unresectable hepatocellular carcinoma conversion therapy: a prospective, single-arm phase II trial. Neoplasma 70(6):811–818. 10.4149/neo_2023_230806N413 38247334 10.4149/neo_2023_230806N413

[16] Li J, Kong M, Yu G, Wang S, Shi Z, Han H et al (2023) Safety and efficacy of transarterial chemoembolization combined with tyrosine kinase inhibitors and camrelizumab in the treatment of patients with advanced unresectable hepatocellular carcinoma. Front Immunol 14:1188308. 10.3389/fimmu.2023.1188308 37545497 10.3389/fimmu.2023.1188308 PMC10401037

[17] Tai D, Loke K, Gogna A, Kaya NA, Tan SH, Hennedige T et al (2021) Radioembolisation with Y90-resin microspheres followed by nivolumab for advanced hepatocellular carcinoma (CA 209-678): a single arm, single centre, phase 2 trial. Lancet Gastroenterol Hepatol 6(12):1025–1035. 10.1016/S2468-1253(21)00305-8 34695377 10.1016/S2468-1253(21)00305-8

[18] Yu S, Yu M, Keane B, Mauro DM, Helft PR, Harris WP et al (2024) A pilot study of pembrolizumab in combination with Y90 radioembolization in subjects with poor prognosis hepatocellular carcinoma. Oncologist 29(3):270-e413. 10.1093/oncolo/oyad331 38325328 10.1093/oncolo/oyad331 PMC10911903

[19] Li X, Ding X, Liu M, Wang J, Sun W, Teng Y et al (2023) A multicenter prospective study of TACE combined with lenvatinib and camrelizumab for hepatocellular carcinoma with portal vein tumor thrombus. Cancer Med 12(16):16805–16814. 10.1002/cam4.6302 37387602 10.1002/cam4.6302 PMC10501288

[20] Lai Z, He M, Bu X, Xu Y, Huang Y, Wen D et al (2022) Lenvatinib, toripalimab plus hepatic arterial infusion chemotherapy in patients with high-risk advanced hepatocellular carcinoma: a biomolecular exploratory, phase II trial. Eur J Cancer 174:68–77. 10.1016/j.ejca.2022.07.005 35981413 10.1016/j.ejca.2022.07.005

[21] He M, Huang Y, Du Z, Lai Z, Ouyang H, Shen J et al (2023) Lenvatinib, toripalimab plus FOLFOX chemotherapy in hepatocellular carcinoma patients with extrahepatic metastasis: a biomolecular exploratory, phase II trial (LTSC). Clin Cancer Res 29(24):5104–5115. 10.1158/1078-0432.CCR-23-0060 37819944 10.1158/1078-0432.CCR-23-0060

[22] Cai M, Huang W, Liang W, Guo Y, Liang L, Lin L et al (2024) Lenvatinib, sintilimab plus transarterial chemoembolization for advanced stage hepatocellular carcinoma: a phase II study. Liver Int 44(4):920–930. 10.1111/liv.15831 38291865 10.1111/liv.15831

[23] Cai H, Chen S, Tang S, Xiao Y, Shi F, Wu Z et al (2025) Lenvatinib and tislelizumab versus atezolizumab and bevacizumab in combination with TAE-HAIC for unresectable hepatocellular carcinoma with high tumor burden: a multicenter retrospective cohort study. Cancer Immunol Immunother 74(3):88. 10.1007/s00262-025-03942-3 39891746 10.1007/s00262-025-03942-3 PMC11787109

[24] Shen L, Cao F, Liu Y, Nuerhashi G, Lin L, Tan H et al (2025) Hepatic artery infusion of FOLFOX chemotherapy plus camrelizumab combined with sorafenib for advanced hepatocellular carcinoma in Barcelona clinic liver cancer stage C (Double-IA-001): a phase II trial. BMC Med 23(1):275. 10.1186/s12916-025-04110-1 40346494 10.1186/s12916-025-04110-1 PMC12065160

[25] Chen S, Shi F, Wu Z, Wang L, Cai H, Ma P et al (2023) Hepatic arterial infusion chemotherapy plus lenvatinib and tislelizumab with or without transhepatic arterial embolization for unresectable hepatocellular carcinoma with portal vein tumor thrombus and high tumor burden: a multicenter retrospective study. J Hepatocell Carcinoma 10:1209–1222. 10.2147/JHC.S417550 37533600 10.2147/JHC.S417550 PMC10390715

[26] Chen Y, Zhang J, Hu W, Li X, Sun K, Shen Y et al (2024) Envafolimab plus lenvatinib and transcatheter arterial chemoembolization for unresectable hepatocellular carcinoma: a prospective, single-arm, phase II study. Signal Transduct Target Ther 9(1):280. 10.1038/s41392-024-01991-1 39384742 10.1038/s41392-024-01991-1 PMC11464841

[27] Zhao Z, Jiang X, Wen S, Hao Y (2024) Efficiency and safety of HAIC combined with lenvatinib and tislelizumab for advanced hepatocellular carcinoma with high tumor burden: a multicenter propensity score matching analysis. Front Pharmacol 15:1499269. 10.3389/fphar.2024.1499269 39840082 10.3389/fphar.2024.1499269 PMC11747745

[28] Ren Y, Li Y, Cao M, Tang Y, Yuan F, Yang G et al (2024) Efficacy and safety of low-dose cyclophosphamide combined with lenvatinib, pembrolizumab and TACE for unresectable hepatocellular carcinoma: a single-center, prospective, single-arm clinical trial. Chin J Cancer Res 36(2):114–123. 10.21147/j.issn.1000-9604.2024.02.02 38751440 10.21147/j.issn.1000-9604.2024.02.02 PMC11090797

[29] Gao W, Pan Z-L, Zhao X-H, Yang L, Cao J-B, Li D-Y et al (2025) Donafenib and sintilimab combined with hepatic arterial infusion chemotherapy for unresectable hepatocellular carcinoma: a prospective, single-arm phase II trial (DoHAICs study). eClinicalMedicine 83:103217. 10.1016/j.eclinm.2025.103217 40475000 10.1016/j.eclinm.2025.103217 PMC12138396

[30] Zhang T-Q, Geng Z-J, Zuo M-X, Li J-B, Huang J-H, Huang Z-L et al (2023) Camrelizumab (a PD-1 inhibitor) plus apatinib (an VEGFR-2 inhibitor) and hepatic artery infusion chemotherapy for hepatocellular carcinoma in Barcelona clinic liver cancer stage C (TRIPLET): a phase II study. Signal Transduct Target Ther 8(1):413. 10.1038/s41392-023-01663-6 37884523 10.1038/s41392-023-01663-6 PMC10603153

[31] Li J, Bai Y, Xiong F, Liu X, Hu J, Zhang G et al (2025) Atezolizumab plus bevacizumab combined with or without transarterial chemoembolization in the treatment of advanced hepatocellular carcinoma: a single-center retrospective study. J Hepatocell Carcinoma 12:973–984. 10.2147/JHC.S515453 40395491 10.2147/JHC.S515453 PMC12090845

[32] Duan X, Li H, Kuang D, Chen P, Zhang K, Li Y et al (2023) Transcatheter arterial chemoembolization plus apatinib with or without camrelizumab for unresectable hepatocellular carcinoma: a multicenter retrospective cohort study. Hepatol Int 17(4):915–926. 10.1007/s12072-023-10519-8 37012542 10.1007/s12072-023-10519-8 PMC10386927

[33] Zhu D, Ma K, Yang W, Zhou H-F, Shi Q, Ren J-W et al (2022) Transarterial chemoembolization plus apatinib with or without camrelizumab for unresected hepatocellular carcinoma: a two-center propensity score matching study. Front Oncol 12:1057560. 10.3389/fonc.2022.1057560 36439471 10.3389/fonc.2022.1057560 PMC9685301

[34] Liu H, Yu Q, Gu T, Qu P, Ma X, Zhou S et al (2023) Transarterial chemoembolization plus apatinib with or without camrelizumab for the treatment of advanced HBV-related hepatocellular carcinoma. J Gastrointest Liver Dis 32(2):182–189. 10.15403/jgld-4667 10.15403/jgld-4667 37345608

[35] Zhao Y, Wen S, Xue Y, Dang Z, Nan Z, Wang D et al (2024) Transarterial chemoembolization combined with lenvatinib plus tislelizumab for unresectable hepatocellular carcinoma: a multicenter cohort study. Front Immunol 15:1449663. 10.3389/fimmu.2024.1449663 39411718 10.3389/fimmu.2024.1449663 PMC11473327

[36] Wu F-D, Zhou H-F, Yang W, Zhu D, Wu B-F, Shi H-B et al (2025) Transarterial chemoembolization combined with lenvatinib and sintilimab versus lenvatinib alone in intermediate-advanced hepatocellular carcinoma. World J Gastrointest Oncol 17(1):96267. 10.4251/wjgo.v17.i1.96267 39817120 10.4251/wjgo.v17.i1.96267 PMC11664616

[37] Zheng Y, Xiang Y, Shi H, Lin Z, Cheng S, Zhu J (2024) Transarterial chemoembolization combined with atezolizumab plus bevacizumab versus transarterial chemoembolization alone in intermediate‐stage hepatocellular carcinoma: a multicenter retrospective study. J Hepatocell Carcinoma 11:1079–1093. 10.2147/JHC.S461630 38882440 10.2147/JHC.S461630 PMC11180435

[38] Zhao C, Xiang Z, Li M, Wang H, Liu H, Yan H et al (2023) Transarterial chemoembolization combined with atezolizumab plus bevacizumab or lenvatinib for unresectable hepatocellular carcinoma: a propensity score matched study. J Hepatocell Carcinoma 10:1195–1206. 10.2147/JHC.S418256 37521029 10.2147/JHC.S418256 PMC10386869

[39] Chen S, Shuangyan T, Shi F, Cai H, Wu Z, Wang L et al (2024) TACE plus lenvatinib and tislelizumab for intermediate-stage hepatocellular carcinoma beyond up-to-11 criteria: a multicenter cohort study. Front Immunol 15:1430571. 10.3389/fimmu.2024.1430571 39131156 10.3389/fimmu.2024.1430571 PMC11310062

[40] Xiang Z, Li G, Mu L, Wang H, Zhou C, Yan H et al (2023) TACE combined with lenvatinib and camrelizumab for unresectable multiple nodular and large hepatocellular carcinoma (>5 cm). Technol Cancer Res Treat 22:15330338231200320. 10.1177/15330338231200320 37723998 10.1177/15330338231200320 PMC10510362

[41] Tang Z, Bai T, Wei T, Wang X, Chen J, Ye J et al (2024) TACE combined lenvatinib plus camrelizumab versus TACE alone in efficacy and safety for unresectable hepatocellular carcinoma: a propensity score-matching study. BMC Cancer 24(1):717. 10.1186/s12885-024-12484-3 38862932 10.1186/s12885-024-12484-3 PMC11165855

[42] Sun B, Zhang L, Sun T, Ren Y, Cao Y, Zhang W et al (2022) Safety and efficacy of lenvatinib combined with camrelizumab plus transcatheter arterial chemoembolization for unresectable hepatocellular carcinoma: a two-center retrospective study. Front Oncol 12:982948. 10.3389/fonc.2022.982948 36172158 10.3389/fonc.2022.982948 PMC9511022

[43] Jin Z-C, Zhong B-Y, Chen J-J, Zhu H-D, Sun J-H, Yin G-W et al (2023) Real-world efficacy and safety of TACE plus camrelizumab and apatinib in patients with HCC (CHANCE2211): a propensity score matching study. Eur Radiol 33(12):8669–8681. 10.1007/s00330-023-09754-2 37368105 10.1007/s00330-023-09754-2 PMC10667391

[44] Chen S, Wu Z, Shi F, Mai Q, Wang L, Wang F et al (2022) Lenvatinib plus TACE with or without pembrolizumab for the treatment of initially unresectable hepatocellular carcinoma harbouring PD-L1 expression: a retrospective study. J Cancer Res Clin Oncol 148(8):2115–2125. 10.1007/s00432-021-03767-4 34453221 10.1007/s00432-021-03767-4 PMC9293824

[45] Lang M, Gan L, Ren S, Han R, Ma X, Li G et al (2023) Lenvatinib plus sintilimab with or without transarterial chemoembolization for intermediate or advanced stage hepatocellular carcinoma: a propensity score-matching cohort study. Am J Cancer Res 13(6):2540–2553 37424821 PMC10326569

[46] Cao F, Shi C, Zhang G, Luo J, Zheng J, Hao W (2023) Improved clinical outcomes in advanced hepatocellular carcinoma treated with transarterial chemoembolization plus atezolizumab and bevacizumab: a bicentric retrospective study. BMC Cancer 23(1):873. 10.1186/s12885-023-11389-x 37718456 10.1186/s12885-023-11389-x PMC10506240

[47] Li Y, Guo J, Liu W, Pang H, Song Y, Wu S et al (2024) Hepatic artery infusion chemotherapy combined with camrelizumab plus rivoceranib for hepatocellular carcinoma with portal vein tumor thrombosis: a multicenter propensity score-matching analysis. Hepatol Int 18(4):1286–1298. 10.1007/s12072-024-10672-8 38717693 10.1007/s12072-024-10672-8 PMC11297837

[48] Zuo M, Cao Y, Yang Y, Zheng G, Li D, Shao H et al (2024) Hepatic arterial infusion chemotherapy plus camrelizumab and apatinib for advanced hepatocellular carcinoma. Hepatol Int 18(5):1486–1498. 10.1007/s12072-024-10690-6 38961006 10.1007/s12072-024-10690-6 PMC11461759

[49] Wu J, Zeng J, Wang H, Huo Z, Hou X, He D (2023) Efficacy and safety of transarterial chemoembolization combined with lenvatinib and camrelizumab in patients with BCLC-defined stage C hepatocellular carcinoma. Front Oncol 13:1244341. 10.3389/fonc.2023.1244341 37916160 10.3389/fonc.2023.1244341 PMC10616839

[50] Guo Z, Zhu H, Zhang X, Huang L, Wang X, Shi H et al (2022) The efficacy and safety of conventional transcatheter arterial chemoembolization combined with PD-1 inhibitor and anti-angiogenesis tyrosine kinase inhibitor treatment for patients with unresectable hepatocellular carcinoma: a real-world comparative study. Front Oncol 12:941068. 10.3389/fonc.2022.941068 36248989 10.3389/fonc.2022.941068 PMC9558003

[51] Lu H, Liang B, Xia X, Zheng C (2023) Efficacy and safety analysis of TACE + Donafenib + Toripalimab versus TACE + Sorafenib in the treatment of unresectable hepatocellular carcinoma: a retrospective study. BMC Cancer 23(1):1033. 10.1186/s12885-023-11535-5 37880661 10.1186/s12885-023-11535-5 PMC10599044

[52] Qu W-F, Ding Z-B, Qu X-D, Tang Z, Zhu G-Q, Fu X-T et al (2022) Conversion therapy for initially unresectable hepatocellular carcinoma using a combination of toripalimab, lenvatinib plus TACE: real-world study. BJS Open 6(5):zrac114. 10.1093/bjsopen/zrac114 36125345 10.1093/bjsopen/zrac114 PMC9499852

[53] Ju S, Zhou C, Yang C, Wang C, Liu J, Wang Y et al (2021) Apatinib plus camrelizumab with/without chemoembolization for hepatocellular carcinoma: a real-world experience of a single center. Front Oncol 11:835889. 10.3389/fonc.2021.835889 35174073 10.3389/fonc.2021.835889 PMC8841670

[54] Jiang J, Zhang H, Lai J, Zhang S, Ou Y, Fu Y et al (2024) Efficacy and safety of transarterial chemoembolization plus lenvatinib with or without tislelizumab as the first-line treatment for unresectable hepatocellular carcinoma: a propensity score matching analysis. J Hepatocell Carcinoma 11:1607–1622. 10.2147/JHC.S472286 39206422 10.2147/JHC.S472286 PMC11352531

[55] Jiang X, Wang P, Su K, Li H, Chi H, Wang F et al (2024) Camrelizumab combined with transcatheter arterial chemoembolization and sorafenib or lenvatinib for unresectable hepatocellular carcinoma: a multicenter, retrospective study. Ann Hepatol 30(2):101578. 10.1016/j.aohep.2024.101578 39276984 10.1016/j.aohep.2024.101578

[56] Pinato DJ, Murray SM, Forner A, Kaneko T, Fessas P, Toniutto P et al (2021) Trans-arterial chemoembolization as a loco-regional inducer of immunogenic cell death in hepatocellular carcinoma: implications for immunotherapy. J Immunother Cancer. 10.1136/jitc-2021-003311 34593621 10.1136/jitc-2021-003311 PMC8487214

[57] Cai M, Huang W, Huang J, Shi W, Guo Y, Liang L et al (2022) Transarterial chemoembolization combined with lenvatinib plus PD-1 inhibitor for advanced hepatocellular carcinoma: a retrospective cohort study. Front Immunol 13:848387. 10.3389/fimmu.2022.848387 35300325 10.3389/fimmu.2022.848387 PMC8921060

[58] Lee A, Keam SJ (2020) Tislelizumab: first approval. Drugs 80(6):617–624. 10.1007/s40265-020-01286-z 32185681 10.1007/s40265-020-01286-z

[59] Zhang T, Song X, Xu L, Ma J, Zhang Y, Gong W et al (2018) The binding of an anti-PD-1 antibody to FcgammaRIota has a profound impact on its biological functions. Cancer Immunol Immunother 67(7):1079–1090. 10.1007/s00262-018-2160-x 29687231 10.1007/s00262-018-2160-x PMC6006217

[60] Liang Z, Lei J, Li H, Dai H, Zhang Y, Wang F et al (2025) HBV reactivation and prognosis after systemic therapy in HCC with undetectable HBV DNA: a multicenter retrospective study. Sci Rep 15(1):30204. 10.1038/s41598-025-13406-4 40826083 10.1038/s41598-025-13406-4 PMC12361520

[61] Choi WM, Lee D, Shim JH, Kim KM, Lim YS, Lee HC et al (2020) Effectiveness and safety of nivolumab in Child-Pugh B patients with hepatocellular carcinoma: a real-world cohort study. Cancers (Basel). 10.3390/cancers12071968 32698355 10.3390/cancers12071968 PMC7409289

[62] Xie E, Yeo YH, Scheiner B, Zhang Y, Hiraoka A, Tantai X et al (2023) Immune checkpoint inhibitors for Child-Pugh class B advanced hepatocellular carcinoma: a systematic review and meta-analysis. JAMA Oncol 9(10):1423–1431. 10.1001/jamaoncol.2023.3284 37615958 10.1001/jamaoncol.2023.3284 PMC10450588

