# Supplementary material for: Comparative efficacy of combination regimens based on interventional therapy and immune checkpoint inhibitors (ICIs) in patients with intermediate- and advanced-stage hepatocellular carcinoma: a systematic review, meta-analysis, and network meta-analysis
Source: Cancer Immunol Immunother. 2026 Feb 7;75(3):67. doi: 10.1007/s00262-025-04251-5 (PMC12882918; doi:10.1007/s00262-025-04251-5)
Supplement: Supplementary file 1 — Supplementary file1 (PDF 9100 kb) [file 262_2025_4251_MOESM1_ESM.pdf]

Supplementary materials

Fig.S1	Risk of bias assessment for the included single-arm trials according to the MINORS evaluation criteria	2
Fig.S2	Risk of bias assessments for the included randomised controlled studies according to the Cochrane Collaboration's tool	2
Fig.S3	Pooled efficacy and safety estimates of each combined treatment strategy in single-arm meta-analysis	3-6
Fig.S4	Convergence of the four Markov Chain Monte Carlo (MCMC) chains established by the Brooks-Gelman-Rubin diagnostic feature	7
Fig.S5	Comparative network plots for progression-free survival subgroup analysis	8
Fig.S6	Comparative network plots for overall survival subgroup analysis	9
Fig.S7	Pooled estimates and SUCRA results from the subgroup network meta-analysis for progression-free survival	10
Fig.S8	Pooled estimates and SUCRA results from the subgroup network meta-analysis for overall survival	11
Fig.S9	Pooled estimates and SUCRA results of the network meta-analysis for progression-free survival in the sensitivity analysis including studies with sample sizes greater than 30	12
Table S1	Checklist of the PRISMA extension for network meta-analysis	13-15
Table S2	Literature search strategy	16-18
Table S3	Baseline characteristics of studies included in the network meta-analysis	19-28
Table S4	Risk of bias assessment for the included cohort studies according to the NOS evaluation criteria	29
Table S5	Bayesian ranking results of network meta-analysis	30-32
Table S6	Comparison of the fit goodness between consistency and inconsistency models based on DIC values in network meta-analysis	33
Table S7	Inconsistency analysis of network meta-analysis results	34-36
Table S8	Post-study treatment in follow-up	37-40

Fig.S1 Risk of bias assessment for the included single-arm trials according to the MINORS evaluation criteria.

	Li,2023(1)	Wu,2023(2)	Li,2023(2)	Cai,2023	Chen,2024(2)	Zhang,2023	He,2023	Lai,2022	Tai,2021	Yu,2024	Liu,2023(2)	Ren,2024	Mu, 2025	Gao, 2025	Shen, 2025	
A clearly stated aim	2	2	2	2	2	2	2	2	2	2	2	2	2	2	2	Item 1
Inclusion of consecutive patients	2	2	2	2	2	2	2	2	2	2	2	2	2	2	2	Item 2
Prospective collection of data	2	2	2	2	2	2	2	2	2	2	2	2	2	2	2	Item 3
Endpoints appropriate to the aim of the study	2	2	2	2	2	2	2	2	2	2	2	2	2	2	2	Item 4
Unbiased assessment of the study endpoint	1	2	1	1	1	1	1	1	1	1	1	1	1	1	2	Item 5
Follow-up period appropriate to the aim of the study	2	0	0	0	0	1	0	0	2	0	0	0	0	1	2	Item 6
Loss to follow up less than 5%	2	2	0	0	2	0	0	0	2	0	0	2	0	0	0	Item 7
Prospective calculation of the study size	2	2	0	2	2	2	2	2	2	2	2	2	2	2	2	Item 8
An adequate control group	NA	NA	NA	NA	NA	NA	NA	NA	NA	NA	NA	NA	NA	NA	NA	Item 9*
Contemporary groups	NA	NA	NA	NA	NA	NA	NA	NA	NA	NA	NA	NA	NA	NA	NA	Item 10*
Baseline equivalence of groups	NA	NA	NA	NA	NA	NA	NA	NA	NA	NA	NA	NA	NA	NA	NA	Item 11*
Adequate statistical analyses	NA	NA	NA	NA	NA	NA	NA	NA	NA	NA	NA	NA	NA	NA	NA	Item 12*
**Total scores**	**15**	**14**	**9**	**11**	**13**	**12**	**11**	**11**	**15**	**11**	**11**	**13**	**11**	**12**	**14**	

*For comparative studies only. #Scores of at least 75% were considered high quality with low risk for bias; scores between 50% and 75% were considered medium risk for bias; scores of less than or equal to 50% were considered high risk for bias. For noncomparative studies, the maximum score was 16, while the maximum score for comparative studies was 24. MINORS=methodological index for non-randomised studies. NA=not applicable.

Fig.S2 Risk of bias assessments for the included randomised controlled studies according to the Cochrane Collaboration's tool

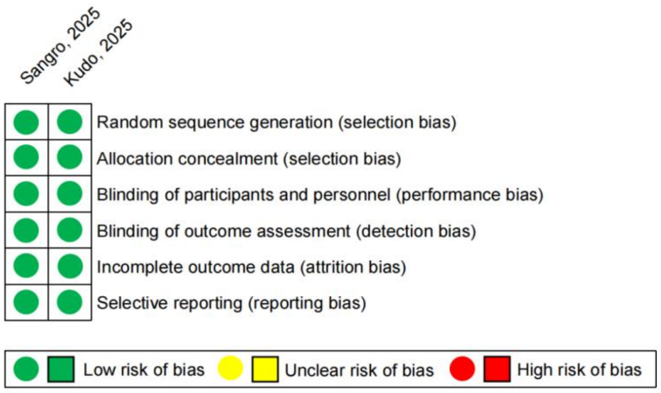

Fig.S3 Pooled efficacy and safety estimates of each combined treatment strategy in single-arm meta-analysis

a

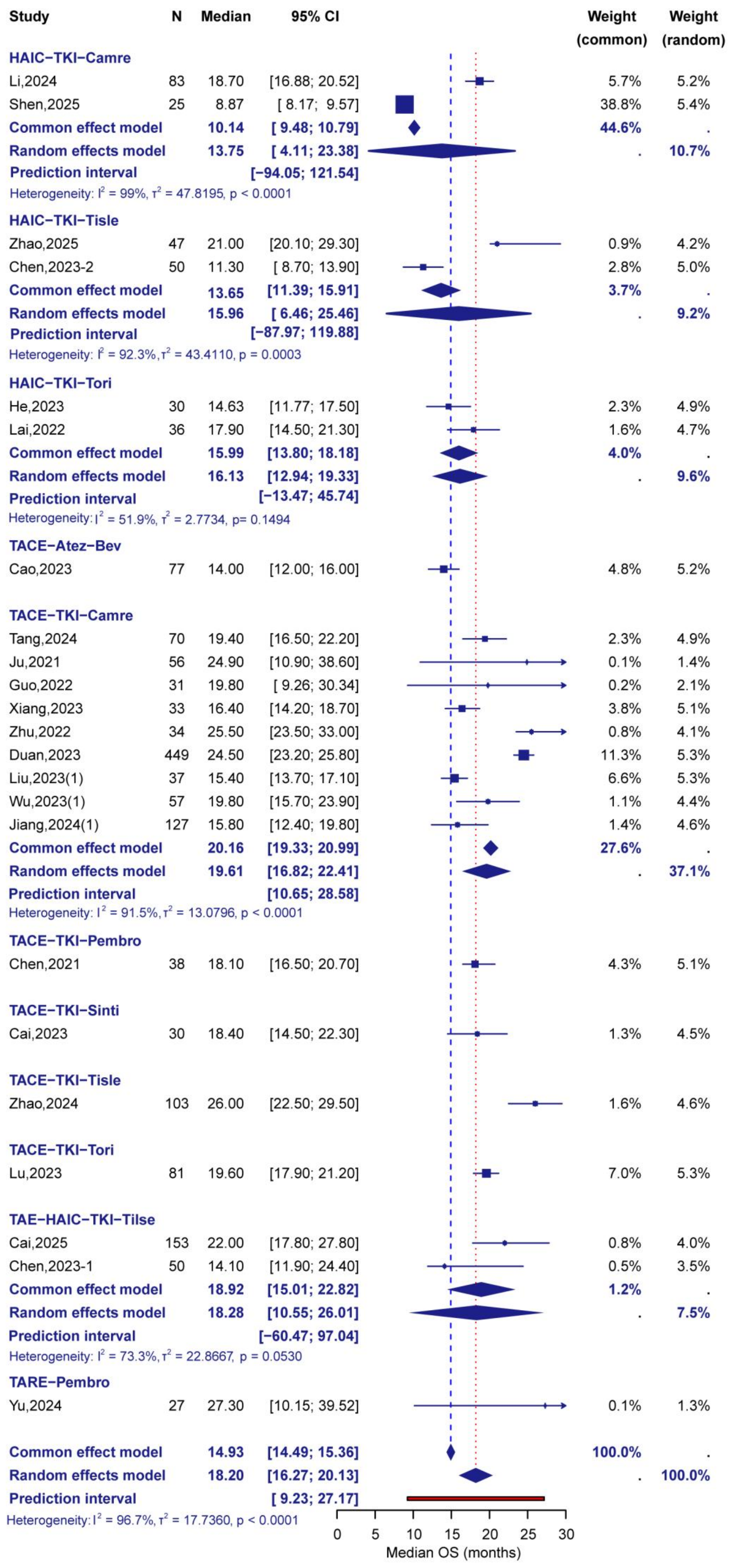

b

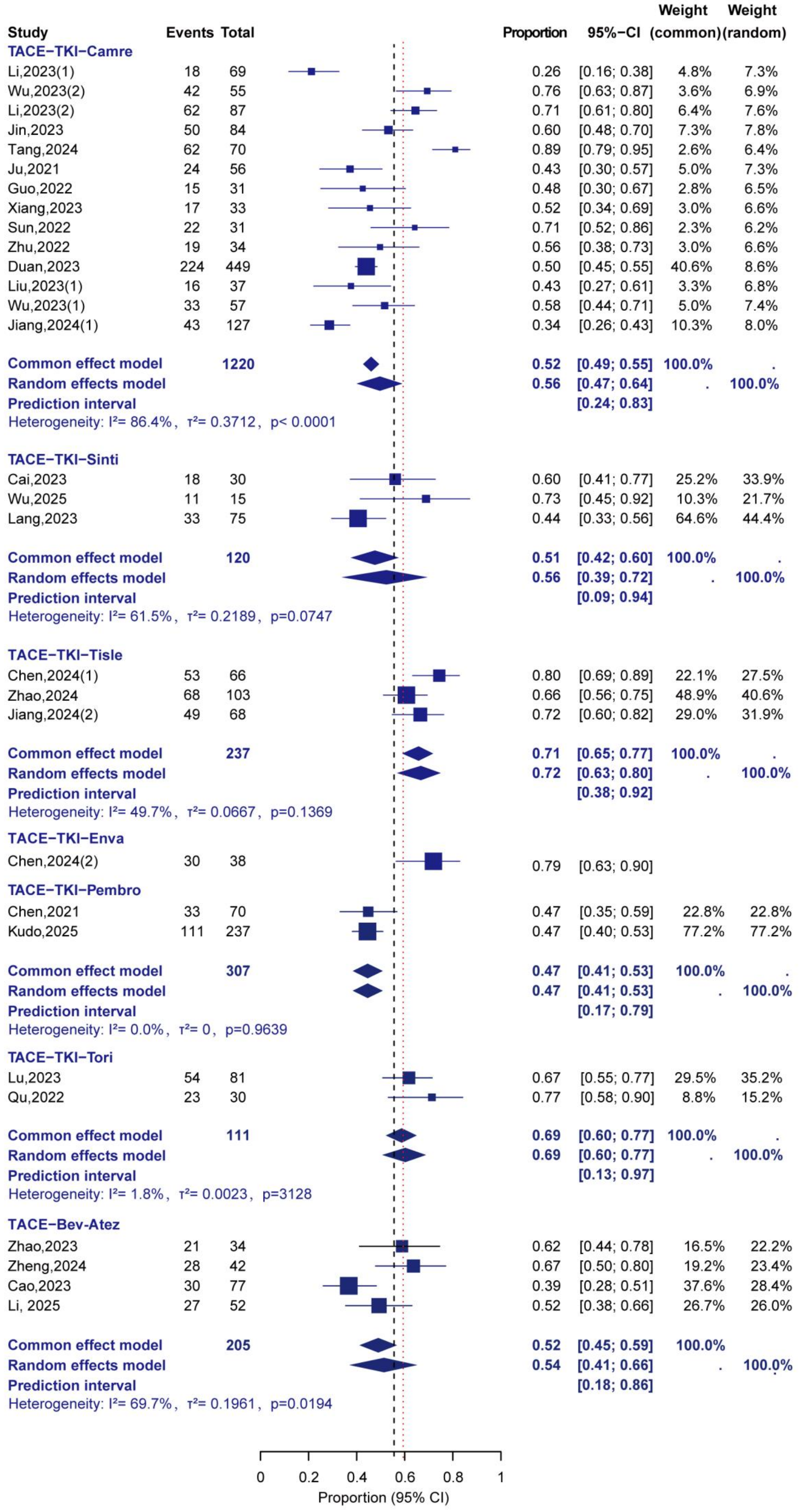

Study Events Total Proportion 95%−CI Weight (common) Weight (random)
TACE−TKI−Camre
Li,2023(1) 18 69 0.26 [0.16; 0.38] 4.8% 7.3%
Wu,2023(2) 42 55 0.76 [0.63; 0.87] 3.6% 6.9%
Li,2023(2) 62 87 0.71 [0.61; 0.80] 6.4% 7.6%
Jin,2023 50 84 0.60 [0.48; 0.70] 7.3% 7.8%
Tang,2024 62 70 0.89 [0.79; 0.95] 2.6% 6.4%
Ju,2021 24 56 0.43 [0.30; 0.57] 5.0% 7.3%
Guo,2022 15 31 0.48 [0.30; 0.67] 2.8% 6.5%
Xiang,2023 17 33 0.52 [0.34; 0.69] 3.0% 6.6%
Sun,2022 22 31 0.71 [0.52; 0.86] 2.3% 6.2%
Zhu,2022 19 34 0.56 [0.38; 0.73] 3.0% 6.6%
Duan,2023 224 449 0.50 [0.45; 0.55] 40.6% 8.6%
Liu,2023(1) 16 37 0.43 [0.27; 0.61] 3.3% 6.8%
Wu,2023(1) 33 57 0.58 [0.44; 0.71] 5.0% 7.4%
Jiang,2024(1) 43 127 0.34 [0.26; 0.43] 10.3% 8.0%
Common effect model 1220 0.52 [0.49; 0.55] 100.0% .
Random effects model 0.56 [0.47; 0.64] . 100.0%
Prediction interval [0.24; 0.83]
Heterogeneity: I²= 86.4%, τ²= 0.3712, p< 0.0001
TACE−TKI−Sinti
Cai,2023 18 30 0.60 [0.41; 0.77] 25.2% 33.9%
Wu,2025 11 15 0.73 [0.45; 0.92] 10.3% 21.7%
Lang,2023 33 75 0.44 [0.33; 0.56] 64.6% 44.4%
Common effect model 120 0.51 [0.42; 0.60] 100.0% .
Random effects model 0.56 [0.39; 0.72] . 100.0%
Prediction interval [0.09; 0.94]
Heterogeneity: I²= 61.5%, τ²= 0.2189, p=0.0747
TACE−TKI−Tisle
Chen,2024(1) 53 66 0.80 [0.69; 0.89] 22.1% 27.5%
Zhao,2024 68 103 0.66 [0.56; 0.75] 48.9% 40.6%
Jiang,2024(2) 49 68 0.72 [0.60; 0.82] 29.0% 31.9%
Common effect model 237 0.71 [0.65; 0.77] 100.0% .
Random effects model 0.72 [0.63; 0.80] . 100.0%
Prediction interval [0.38; 0.92]
Heterogeneity: I²= 49.7%, τ²= 0.0667, p=0.1369
TACE−TKI−Enva
Chen,2024(2) 30 38 0.79 [0.63; 0.90]
TACE−TKI−Pembro
Chen,2021 33 70 0.47 [0.35; 0.59] 22.8% 22.8%
Kudo,2025 111 237 0.47 [0.40; 0.53] 77.2% 77.2%
Common effect model 307 0.47 [0.41; 0.53] 100.0% .
Random effects model 0.47 [0.41; 0.53] . 100.0%
Prediction interval [0.17; 0.79]
Heterogeneity: I²= 0.0%, τ²= 0, p=0.9639
TACE−TKI−Tori
Lu,2023 54 81 0.67 [0.55; 0.77] 29.5% 35.2%
Qu,2022 23 30 0.77 [0.58; 0.90] 8.8% 15.2%
Common effect model 111 0.69 [0.60; 0.77] 100.0% .
Random effects model 0.69 [0.60; 0.77] . 100.0%
Prediction interval [0.13; 0.97]
Heterogeneity: I²= 1.8%, τ²= 0.0023, p=3128
TACE−Bev−Atez
Zhao,2023 21 34 0.62 [0.44; 0.78] 16.5% 22.2%
Zheng,2024 28 42 0.67 [0.50; 0.80] 19.2% 23.4%
Cao,2023 30 77 0.39 [0.28; 0.51] 37.6% 28.4%
Li, 2025 27 52 0.52 [0.38; 0.66] 26.7% 26.0%
Common effect model 205 0.52 [0.45; 0.59] 100.0%
Random effects model 0.54 [0.41; 0.66] . 100.0%
Prediction interval [0.18; 0.86]
Heterogeneity: I²= 69.7%, τ²= 0.1961, p=0.0194
0 0.2 0.4 0.6 0.8 1
Proportion (95% CI)

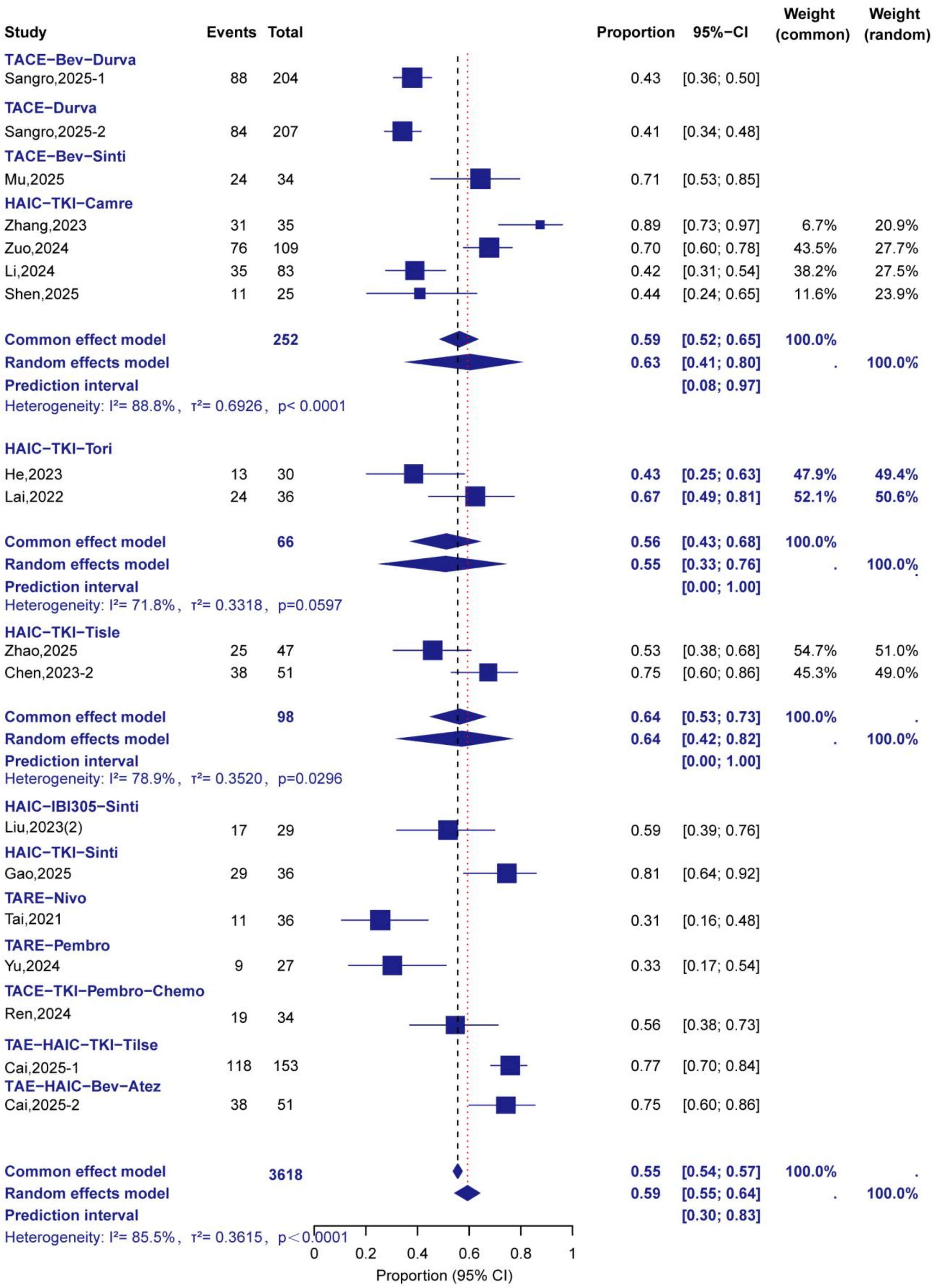
Study Events Total Proportion 95%−CI Weight (common) Weight (random)
TACE−Bev−Durva
Sangro,2025-1 88 204 0.43 [0.36; 0.50]
TACE−Durva
Sangro,2025-2 84 207 0.41 [0.34; 0.48]
TACE−Bev−Sinti
Mu,2025 24 34 0.71 [0.53; 0.85]
HAIC−TKI−Camre
Zhang,2023 31 35 0.89 [0.73; 0.97] 6.7% 20.9%
Zuo,2024 76 109 0.70 [0.60; 0.78] 43.5% 27.7%
Li,2024 35 83 0.42 [0.31; 0.54] 38.2% 27.5%
Shen,2025 11 25 0.44 [0.24; 0.65] 11.6% 23.9%
Common effect model 252 0.59 [0.52; 0.65] 100.0%
Random effects model 0.63 [0.41; 0.80] . 100.0%
Prediction interval [0.08; 0.97]
Heterogeneity: I²= 88.8%，τ²= 0.6926，p< 0.0001
HAIC−TKI−Tori
He,2023 13 30 0.43 [0.25; 0.63] 47.9% 49.4%
Lai,2022 24 36 0.67 [0.49; 0.81] 52.1% 50.6%
Common effect model 66 0.56 [0.43; 0.68] 100.0%
Random effects model 0.55 [0.33; 0.76] . 100.0%
Prediction interval [0.00; 1.00]
Heterogeneity: I²= 71.8%，τ²= 0.3318，p=0.0597
HAIC−TKI−Tisle
Zhao,2025 25 47 0.53 [0.38; 0.68] 54.7% 51.0%
Chen,2023-2 38 51 0.75 [0.60; 0.86] 45.3% 49.0%
Common effect model 98 0.64 [0.53; 0.73] 100.0% .
Random effects model 0.64 [0.42; 0.82] . 100.0%
Prediction interval [0.00; 1.00]
Heterogeneity: I²= 78.9%，τ²= 0.3520，p=0.0296
HAIC−IBI305−Sinti
Liu,2023(2) 17 29 0.59 [0.39; 0.76]
HAIC−TKI−Sinti
Gao,2025 29 36 0.81 [0.64; 0.92]
TARE−Nivo
Tai,2021 11 36 0.31 [0.16; 0.48]
TARE−Pembro
Yu,2024 9 27 0.33 [0.17; 0.54]
TACE−TKI−Pembro−Chemo
Ren,2024 19 34 0.56 [0.38; 0.73]
TAE−HAIC−TKI−Tilse
Cai,2025-1 118 153 0.77 [0.70; 0.84]
TAE−HAIC−Bev−Atez
Cai,2025-2 38 51 0.75 [0.60; 0.86]
Common effect model 3618 0.55 [0.54; 0.57] 100.0% .
Random effects model 0.59 [0.55; 0.64] . 100.0%
Prediction interval [0.30; 0.83]
Heterogeneity: I²= 85.5%，τ²= 0.3615，p＜0.0001
0 0.2 0.4 0.6 0.8 1
Proportion (95% CI)

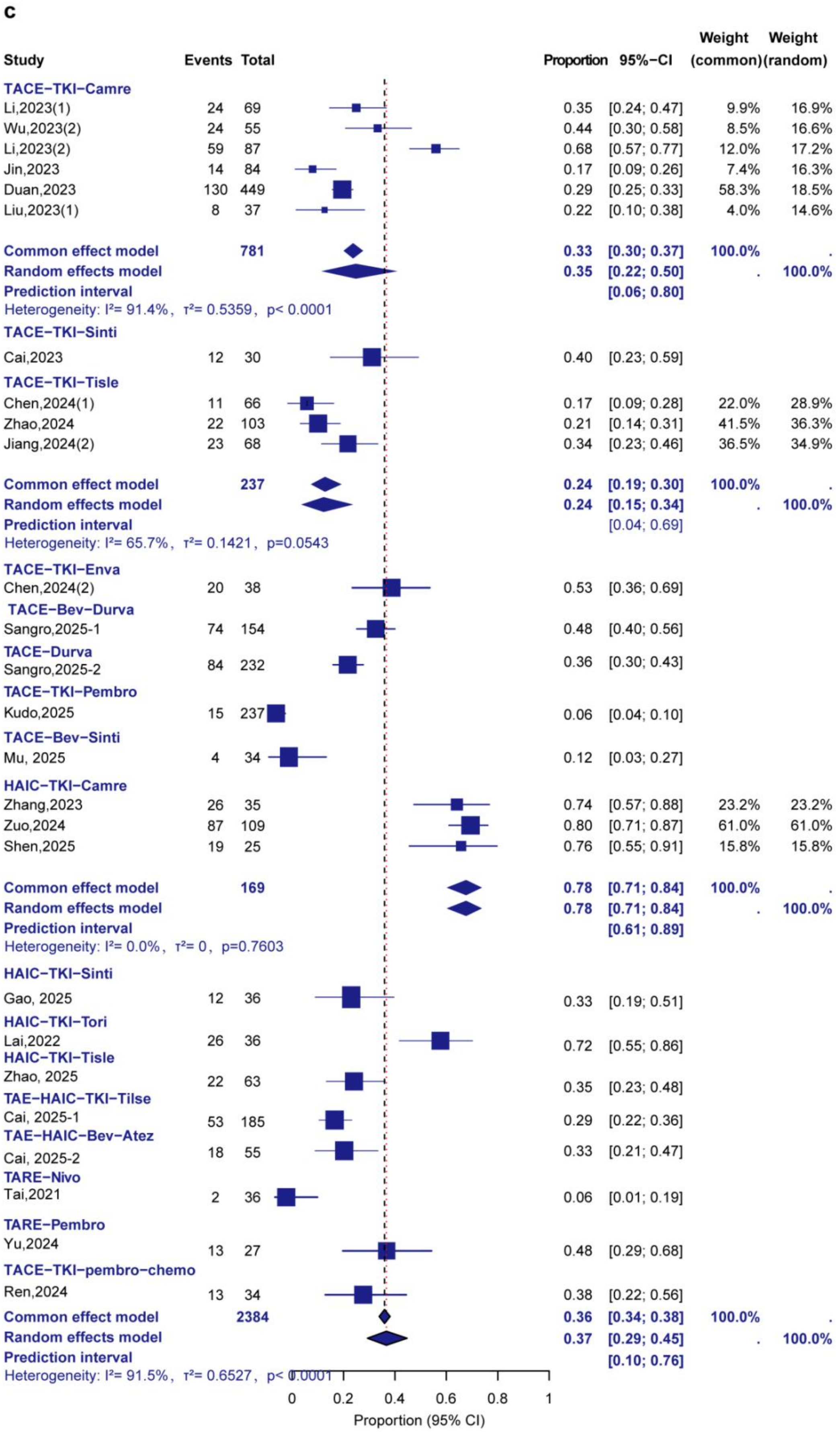

Forest plots show the pooled effect sizes with 95% confidence intervals for (a) median overall survival, (b) objective response rate, and (c) grade ⩾3 adverse events, providing a descriptive overview of different treatment regimens. TACE=transcatheter arterial chemoembolization. HAIC=hepatic arterial infusion chemotherapy. TARE=transarterial radioembolization. TKI=tyrosine-kinase inhibitor. Bev=bevacizumab. Camre=camrelizumab. Pembro=pembrolizumab. Tisle=tislelizumab. Tori=toripalimab. Atez=atezolizumab. Sinti=sintilimab. Envafo=envafolimab. Durva=Durvalumab. Chemo=chemotherapy. Envafo=envafolimab. TARE=transarterial radioembolization. Chemo=chemotherapy.

Fig.S4 Convergence of the four Markov Chain Monte Carlo (MCMC) chains established by the Brooks-Gelman-Rubin diagnostic feature

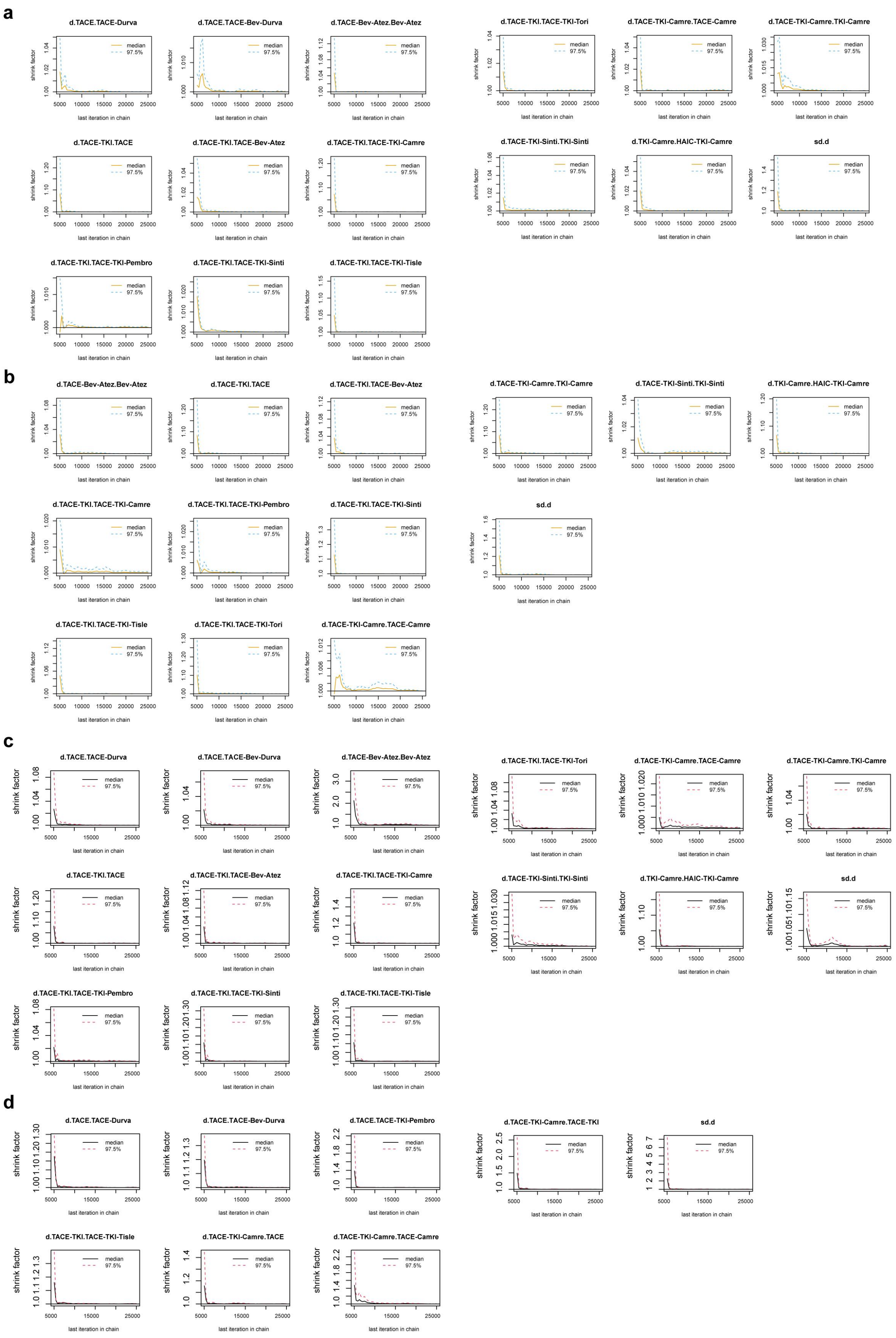

(a)Progression-free survival, (b) Overall survival, (c) Objective response rate, (d) grade ≥3 adverse events. In each Bayesian inference analysis, four independent MCMC chains are generated and running 5,0000 sample iterations per chain simultaneously. TACE=transcatheter arterial chemoembolization. HAIC=hepatic arterial infusion chemotherapy. TKI=tyrosine-kinase inhibitor. Bev=bevacizumab. Camre=camrelizumab. Pembro=pembrolizumab. Tisle=tislelizumab. Tori=toripalimab. Atez=atezolizumab. Sinti=sintilimab. Durva=Durvalumab.

Fig.S5 Comparative network plots for progression-free survival subgroup analysis

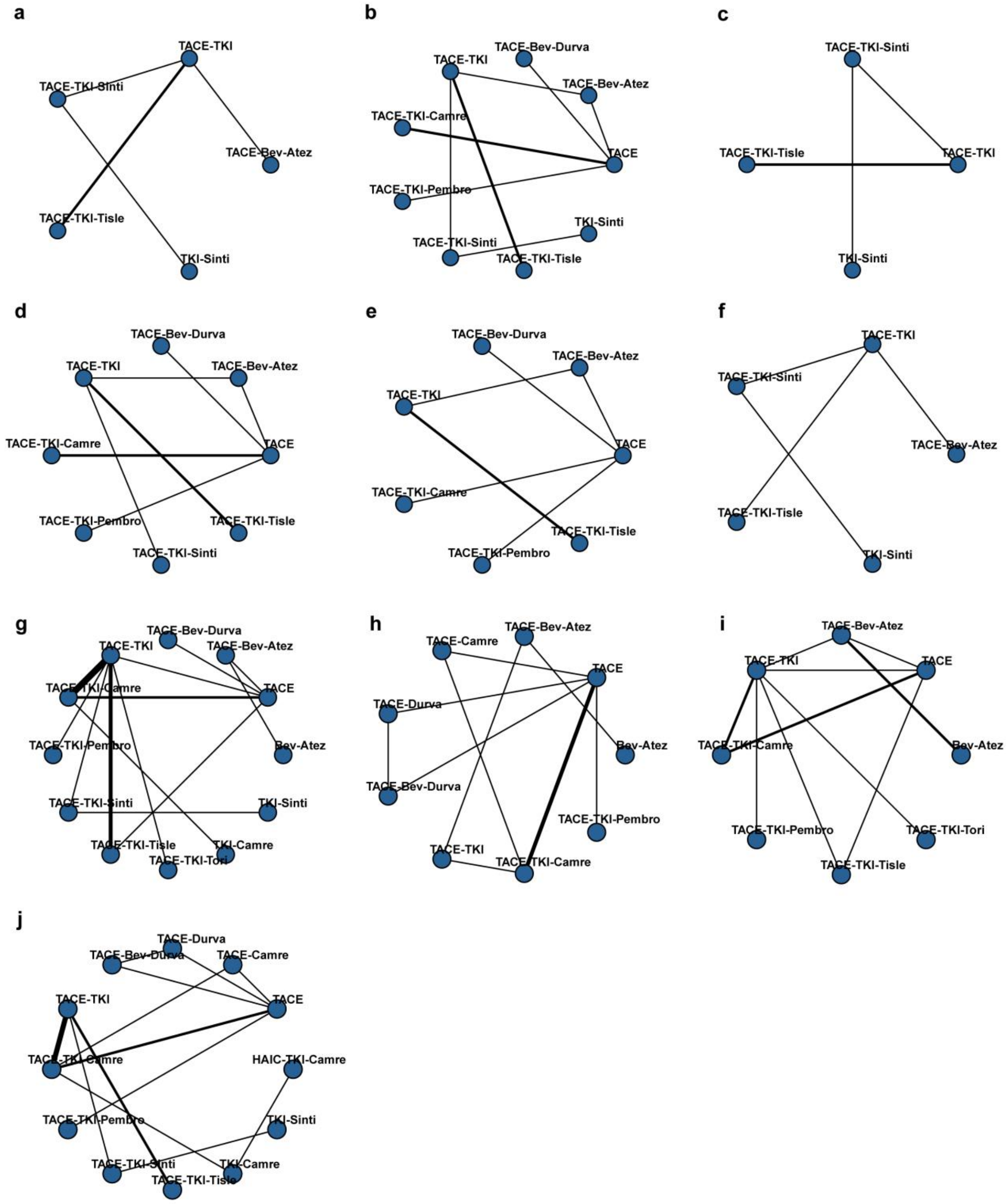

Each network plot represents the comparative connections among treatment regimens within specific subgroups. (a) ECOG PS; (b) Child-Pugh class A; (c) Child-Pugh class B and number of tumors; (d) HBV status; (e) AFP level; (f) Metastasis status and BCLC stage; (g) TACE type: cTACE only; (h) TACE type: cTACE or dTACE; (i) No Prior treatment; (j) Prior treatment. The width of each edge is proportionally weighted according to the number of comparisons. TACE=transcatheter arterial chemoembolization. cTACE=conventional TACE. dTACE=drug-eluting bead TACE. HAIC=hepatic arterial infusion chemotherapy. TKI=tyrosine-kinase inhibitor. Bev=bevacizumab. Camre=camrelizumab. Pembro=pembrolizumab. Tisle=tislelizumab. Tori=toripalimab. Atez=atezolizumab. Sinti=sintilimab. Durva=Durvalumab. ECOG PS=eastern cooperative oncology group performance status score. HBV=hepatitis B virus. AFP=alpha-Fetal Protein. BCLC = Barcelona Clinic Liver Cancer staging system.

Fig.S6 Comparative network plots for overall survival subgroup analysis

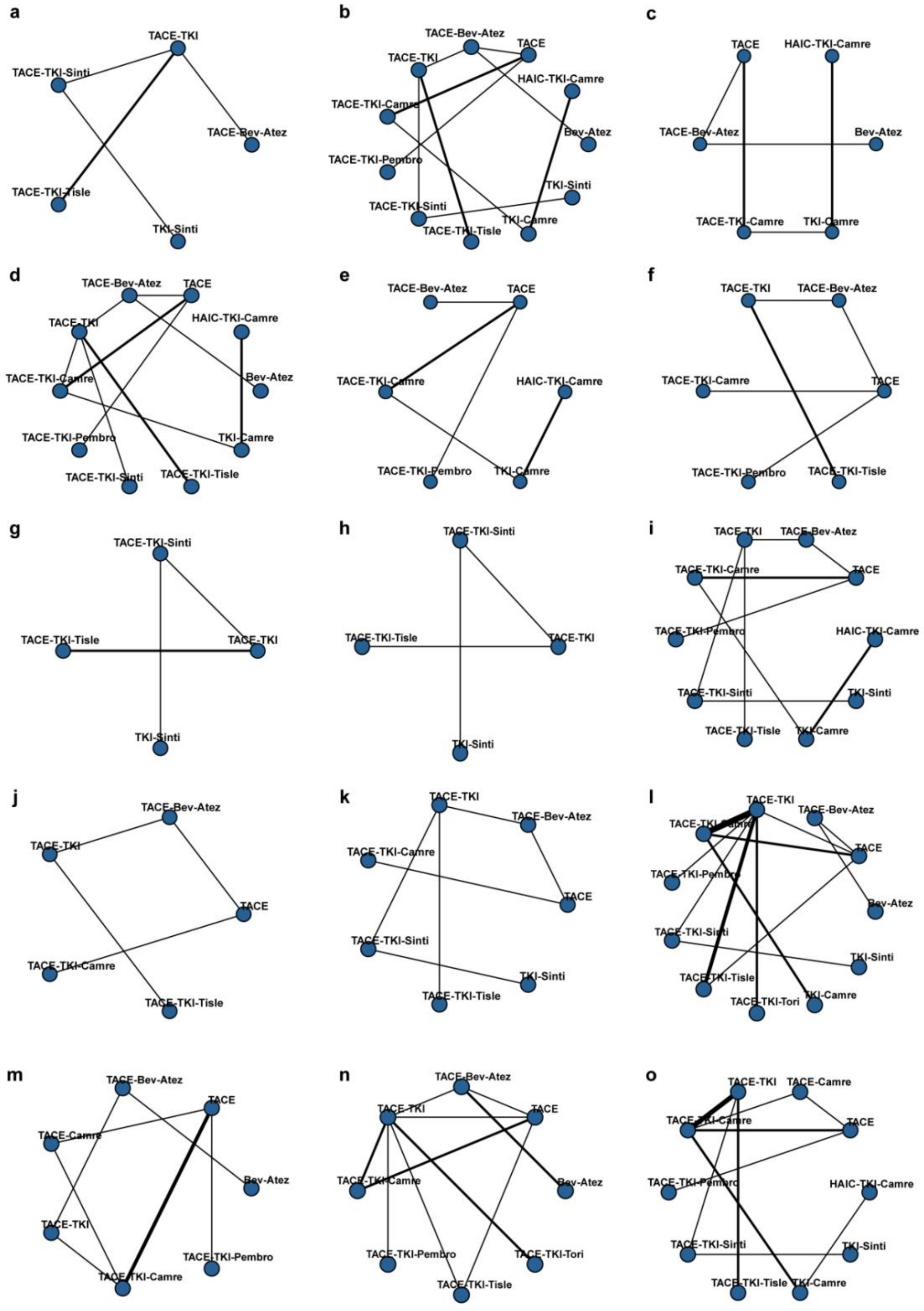

Each network plot represents the comparative connections among treatment regimens within specific subgroups. (a) ECOG PS; (b) Child-Pugh class A; (c) Child-Pugh class B; (d) HBV-positive; (e) HBV-negative; (f) AFP level; (g) number of tumors; (h) BCLC stage B; (i) BCLC stage C; (j) presence of metastasis; (k) absence of metastasis; (l) TACE type: cTACE only; (m) TACE type: cTACE or DEB-TACE; (n) No Prior treatment; (o) Prior treatment. The width of each edge is proportionally weighted according to the number of comparisons. TACE=transcatheter arterial chemoembolization. cTACE=conventional TACE. dTACE=drug-eluting bead TACE. HAIC=hepatic arterial infusion chemotherapy. TKI=tyrosine-kinase inhibitor. Bev=bevacizumab. Camre=camrelizumab. Pembro=pembrolizumab. Tisle=tislelizumab. Tori=toripalimab. Atez=atezolizumab. Sinti=sintilimab. Durva=Durvalumab. ECOG PS=eastern cooperative oncology group performance status score. HBV=hepatitis B virus. AFP=alpha-Fetal Protein. BCLC = Barcelona Clinic Liver Cancer staging system.

Fig.S7 Pooled estimates and SUCRA results from the subgroup network meta-analysis for progression-free survival

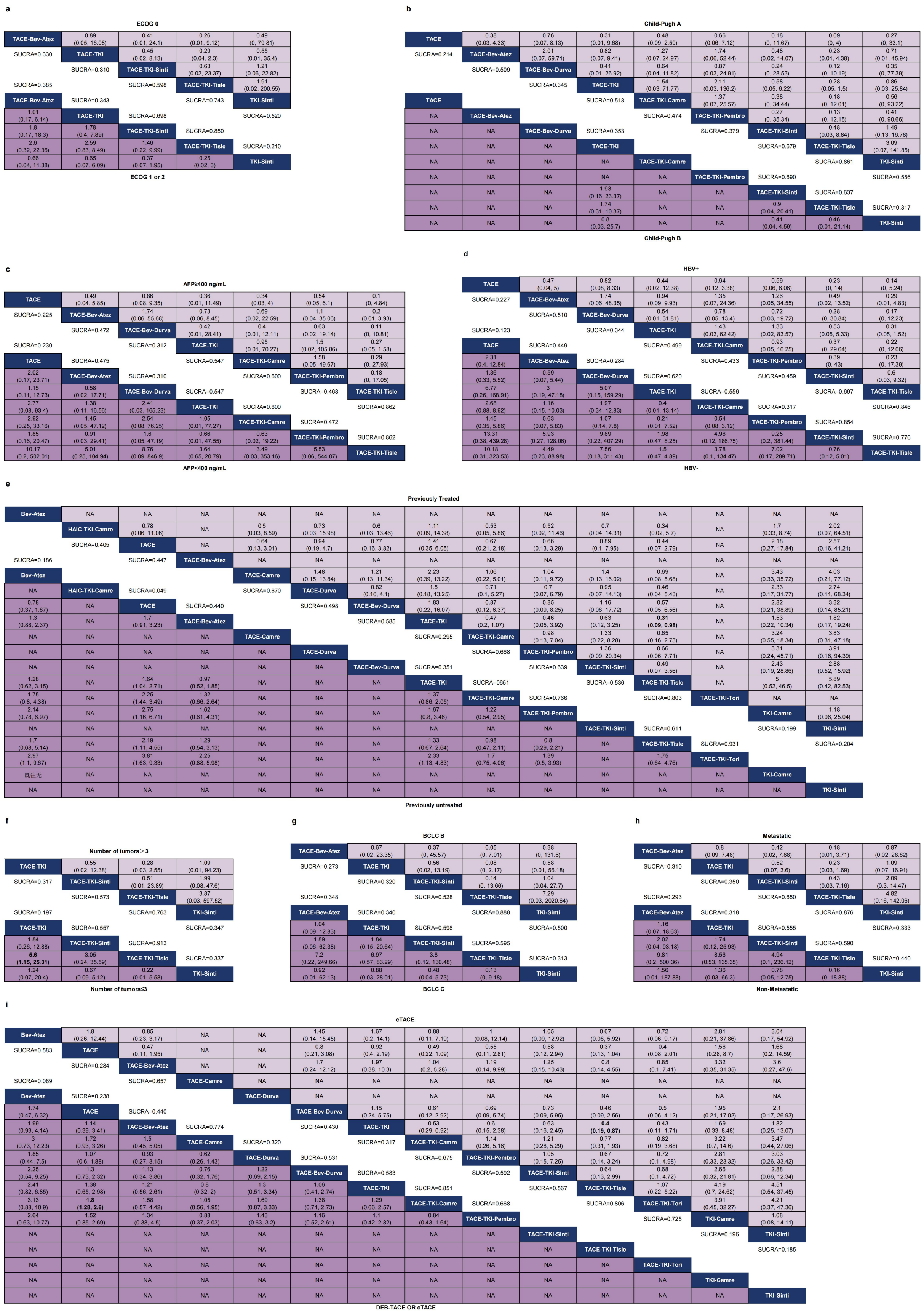

TACE=transcatheter arterial chemoembolization. c-TACE=conventional TACE. DEB-TACE=drug eluting beads TACE. HAIC=hepatic arterial infusion chemotherapy. TKI=tyrosine-kinase inhibitor. Bev=bevacizumab. Camre=camrelizumab. Pembro=pembrolizumab. Tisle=tislelizumab. Tori=toripalimab. Atez=atezolizumab. Sinti=sintilimab. Durva=Durvalumab. ECOG PS=eastern cooperative oncology group performance status score. HBV=hepatitis B virus. AFP=alpha-Fetal Protein. BCLC = Barcelona Clinic Liver Cancer staging system.

Fig.S8 Pooled estimates and SUCRA results from the subgroup network meta-analysis for overall survival

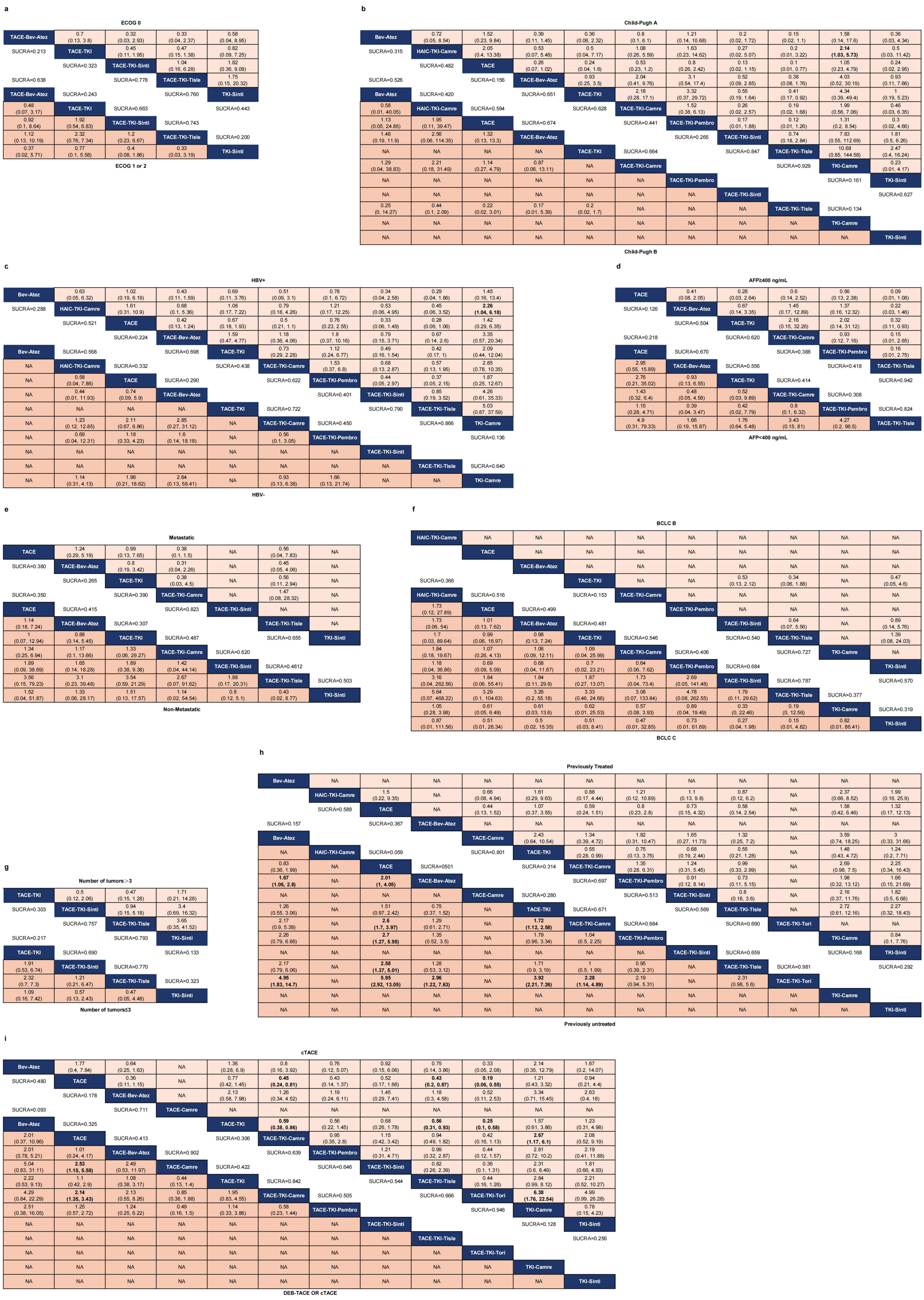

TACE=transcatheter arterial chemoembolization. c-TACE=conventional TACE. DEB-TACE=drug eluting beads TACE. HAIC=hepatic arterial infusion chemotherapy. TKI=tyrosine-kinase inhibitor. Bev=bevacizumab. Camre=camrelizumab. Pembro=pembrolizumab. Tisle=tislelizumab. Tori=toripalimab. Atez=atezolizumab. Sinti=sintilimab. Durva=Durvalumab. ECOG PS=eastern cooperative oncology group performance status score. HBV=hepatitis B virus. AFP=alpha-Fetal Protein. BCLC = Barcelona Clinic Liver Cancer staging system.

Fig.S9. Pooled estimates and SUCRA results of the network meta-analysis for progression-free survival in the sensitivity analysis including studies with sample sizes greater than 30

SUCRA=0.245<br>**Atez-Bev**										
1<br>(0.34, 2.96)	SUCRA=0.185<br>**TACE**									
1.45<br>(0.72, 3.02)	1.46<br>(0.64, 3.34)	SUCRA=0.497<br>**TACE-Atez-Bev**								
1.84<br>(0.45, 7.58)	1.84<br>(0.72, 4.76)	1.27<br>(0.38, 4.32)	SUCRA=0.621<br>**TACE-Camre**							
1.06<br>(0.25, 4.61)	1.07<br>(0.4, 2.8)	0.73<br>(0.2, 2.59)	0.58<br>(0.15, 2.22)	SUCRA=0.270<br>**TACE-Durva**						
1.29<br>(0.3, 5.56)	1.3<br>(0.49, 3.4)	0.89<br>(0.25, 3.14)	0.7<br>(0.18, 2.69)	1.22<br>(0.46, 3.22)	SUCRA=0.408<br>**TACE-Durva-Bev**					
1.17<br>(0.4, 3.51)	1.17<br>(0.71, 1.94)	0.81<br>(0.35, 1.84)	0.64<br>(0.23, 1.71)	1.1<br>(0.38, 3.32)	0.91<br>(0.31, 2.7)	SUCRA=0.302<br>**TACE-TKI**				
2.05<br>(0.69, 6.37)	**2.05<br>(1.29, 3.3)**	1.41<br>(0.6, 3.35)	1.11<br>(0.43, 2.89)	1.93<br>(0.67, 5.73)	1.58<br>(0.55, 4.67)	**1.75<br>(1.2, 2.59)**	SUCRA=0.715<br>**TACE-TKI-Camre**			
1.7<br>(0.48, 6.13)	1.7<br>(0.82, 3.62)	1.17<br>(0.41, 3.41)	0.92<br>(0.29, 3)	1.6<br>(0.48, 5.49)	1.31<br>(0.39, 4.48)	1.45<br>(0.69, 3.1)	0.83<br>(0.38, 1.82)	SUCRA=0.576<br>**TACE-TKI-Pembro**		
2.85<br>(0.85, 9.7)	**2.85<br>(1.43, 5.76)**	1.96<br>(0.74, 5.23)	1.55<br>(0.51, 4.73)	2.68<br>(0.83, 9.04)	2.2<br>(0.68, 7.33)	**2.43<br>(1.37, 4.35)**	1.39<br>(0.71, 2.7)	1.67<br>(0.67, 4.22)	SUCRA=0.874<br>**TACE-TKI-Tisle**	
2.72<br>(0.61, 12.28)	2.73<br>(0.86, 8.66)	1.87<br>(0.5, 7)	1.48<br>(0.35, 6.24)	2.57<br>(0.58, 11.78)	2.1<br>(0.48, 9.57)	2.32<br>(0.82, 6.55)	1.33<br>(0.43, 4.01)	1.61<br>(0.44, 5.7)	0.95<br>(0.29, 3.13)	SUCRA=0.814<br>**TACE-TKI-Tori**

Hazard ratios＜1.00 provides better survival benefits. Bold data indicate a significant difference. TACE=transcatheter arterial chemoembolization. HAIC=hepatic arterial infusion chemotherapy. TKI=tyrosine-kinase inhibitor. Bev=bevacizumab. Camre=camrelizumab. Pembro=pembrolizumab. Tisle=tislelizumab. Tori=toripalimab. Atez=atezolizumab. Sinti=sintilimab. Durva=Durvalumab.

Table S1 Checklist of the PRISMA extension for network meta-analysis.

Section and Topic	Item #	Checklist item	Location where item is reported
**TITLE**			
Title	1	Identify the report as a systematic review.	1
**ABSTRACT**			
Abstract	2	See the PRISMA 2020 for Abstracts checklist.	2
**INTRODUCTION**			
Rationale	3	Describe the rationale for the review in the context of existing knowledge.	3-4
Objectives	4	Provide an explicit statement of the objective(s) or question(s) the review addresses.	4
**METHODS**			
Eligibility criteria	5	Specify the inclusion and exclusion criteria for the review and how studies were grouped for the syntheses.	5
Information sources	6	Specify all databases, registers, websites, organisations, reference lists and other sources searched or consulted to identify studies. Specify the date when each source was last searched or consulted.	5
Search strategy	7	Present the full search strategies for all databases, registers and websites, including any filters and limits used.	5
Selection process	8	Specify the methods used to decide whether a study met the inclusion criteria of the review, including how many reviewers screened each record and each report retrieved, whether they worked independently, and if applicable, details of automation tools used in the process.	5
Data collection process	9	Specify the methods used to collect data from reports, including how many reviewers collected data from each report, whether they worked independently, any processes for obtaining or confirming data from study investigators, and if applicable, details of automation tools used in the process.	5-6
Data items	10a	List and define all outcomes for which data were sought. Specify whether all results that were compatible with each outcome domain in each study were sought (e.g. for all measures, time points, analyses), and if not, the methods used to decide which results to collect.	5-6
	10b	List and define all other variables for which data were sought (e.g. participant and intervention characteristics, funding sources). Describe any assumptions made about any missing or unclear information.	6
Study risk of bias assessment	11	Specify the methods used to assess risk of bias in the included studies, including details of the tool(s) used, how many reviewers assessed each study and whether they worked independently, and if applicable, details of automation tools used in the process.	5-6
Effect measures	12	Specify for each outcome the effect measure(s) (e.g. risk ratio, mean difference) used in the synthesis or presentation of results.	6
Synthesis methods	13a	Describe the processes used to decide which studies were eligible for each synthesis (e.g. tabulating the study intervention characteristics and comparing against the planned groups for each synthesis (item #5)).	6-7

Section and Topic	Item #	Checklist item	Location where item is reported
	13b	Describe any methods required to prepare the data for presentation or synthesis, such as handling of missing summary statistics, or data conversions.	6
	13c	Describe any methods used to tabulate or visually display results of individual studies and syntheses.	6
	13d	Describe any methods used to synthesize results and provide a rationale for the choice(s). If meta-analysis was performed, describe the model(s), method(s) to identify the presence and extent of statistical heterogeneity, and software package(s) used.	6
	13e	Describe any methods used to explore possible causes of heterogeneity among study results (e.g. subgroup analysis, meta-regression).	6
	13f	Describe any sensitivity analyses conducted to assess robustness of the synthesized results.	NA
Reporting bias assessment	14	Describe any methods used to assess risk of bias due to missing results in a synthesis (arising from reporting biases).	6
Certainty assessment	15	Describe any methods used to assess certainty (or confidence) in the body of evidence for an outcome.	NA
**RESULTS**			
Study selection	16a	Describe the results of the search and selection process, from the number of records identified in the search to the number of studies included in the review, ideally using a flow diagram.	6-7
	16b	Cite studies that might appear to meet the inclusion criteria, but which were excluded, and explain why they were excluded.	6-7
Study characteristics	17	Cite each included study and present its characteristics.	7
Risk of bias in studies	18	Present assessments of risk of bias for each included study.	7
Results of individual studies	19	For all outcomes, present, for each study: (a) summary statistics for each group (where appropriate) and (b) an effect estimate and its precision (e.g. confidence/credible interval), ideally using structured tables or plots.	7-8
Results of syntheses	20a	For each synthesis, briefly summarise the characteristics and risk of bias among contributing studies.	6-7
	20b	Present results of all statistical syntheses conducted. If meta-analysis was done, present for each the summary estimate and its precision (e.g. confidence/credible interval) and measures of statistical heterogeneity. If comparing groups, describe the direction of the effect.	7-8
	20c	Present results of all investigations of possible causes of heterogeneity among study results.	9
	20d	Present results of all sensitivity analyses conducted to assess the robustness of the synthesized results.	NA

Section and Topic	Item #	Checklist item	Location where item is reported
Reporting biases	21	Present assessments of risk of bias due to missing results (arising from reporting biases) for each synthesis assessed.	NA
Certainty of evidence	22	Present assessments of certainty (or confidence) in the body of evidence for each outcome assessed.	7-9
**DISCUSSION**			
Discussion	23a	Provide a general interpretation of the results in the context of other evidence.	9-11
	23b	Discuss any limitations of the evidence included in the review.	11
	23c	Discuss any limitations of the review processes used.	11
	23d	Discuss implications of the results for practice, policy, and future research.	11-12
**OTHER INFORMATION**			
Registration and protocol	24a	Provide registration information for the review, including register name and registration number, or state that the review was not registered.	4
	24b	Indicate where the review protocol can be accessed, or state that a protocol was not prepared.	4
	24c	Describe and explain any amendments to information provided at registration or in the protocol.	NA
Support	25	Describe sources of financial or non-financial support for the review, and the role of the funders or sponsors in the review.	NA
Competing interests	26	Declare any competing interests of review authors.	21
Availability of data, code and other materials	27	Report which of the following are publicly available and where they can be found: template data collection forms; data extracted from included studies; data used for all analyses; analytic code; any other materials used in the review.	22

From: Page MJ, McKenzie JE, Bossuyt PM, Boutron I, Hoffmann TC, Mulrow CD, et al. The PRISMA 2020 statement: an updated guideline for reporting systematic reviews. BMJ 2021;372:n71. doi: 10.1136/bmj.n71

Database	Period of search	#	Search strategy
PubMed	1946 to January 2025	1	"liver neoplasms"[MeSH Terms]
		2	(("hepat*"[Title/Abstract] OR "liver"[Title/Abstract]) AND ("carcinom*"[Title/Abstract] OR "neoplasm*"[Title/Abstract] OR "cancer*"[Title/Abstract] OR "malign*"[Title/Abstract] OR "tumor*"[Title/Abstract] OR "tumour*"[Title/Abstract])) OR "HCC"[Title/Abstract]
		3	#1 OR #2
		4	"Immune Checkpoint Inhibitors" [MeSH Terms]
		5	"immune checkpoint inhibitor*"[Title/Abstract] OR "ICI"[Title/Abstract] OR "PD-1"[Title/Abstract] OR "PD-L1"[Title/Abstract] OR "CTLA4"[Title/Abstract] OR "Atezolizumab"[Title/Abstract] OR "Tecentriq"[Title/Abstract] OR "Sintilimab"[Title/Abstract] OR "Camrelizumab"[Title/Abstract] OR "Pembrolizumab"[Title/Abstract] OR "Keytruda"[Title/Abstract] OR "Nivolumab"[Title/Abstract] OR "Opdivo"[Title/Abstract] OR "Toripalimab"[Title/Abstract] OR "Tremelimumab"[Title/Abstract] OR "Imjudo"[Title/Abstract] OR "Ipilimumab"[Title/Abstract] OR "Yervoy"[Title/Abstract] OR "Durvalumab"[Title/Abstract] OR "Imfinzi"[Title/Abstract] OR "Penpulimab"[Title/Abstract] OR "Tislelizumab"[Title/Abstract] OR "Envafolimab"[Title/Abstract] OR "Cadonilimab"[Title/Abstract]
		6	#4 OR #5
		7	"Chemoembolization, Therapeutic"[Mesh]
		8	(("transcatheter"[Title/Abstract] OR "transarterial"[Title/Abstract]) AND "chemoemboli*"[Title/Abstract]) OR "TACE"[Title/Abstract] OR "transcatheter arterial embolization"[Title/Abstract] OR "TAE"[Title/Abstract] OR ("hepatic artery infusion chemotherapy"[Title/Abstract] OR "HAIC"[Title/Abstract])
		9	#7 OR #8
		10	#3 AND #6 AND #9
Embase	1974 to January 2025	1	'liver tumor'/exp
		2	((hepat*:ab,ti OR liver:ab,ti) AND (carcinom*:ab,ti OR neoplasm*:ab,ti OR cancer*:ab,ti OR tumor*:ab,ti OR tumour*:ab,ti OR malign*:ab,ti)) OR hcc:ab,ti
		3	#1 OR #2
		4	'immune checkpoint inhibitors'/exp
		5	'immune checkpoint inhibitor*':ab,ti OR ici:ab,ti OR 'pd-1':ab,ti OR 'pd-l1':ab,ti OR ctla4:ab,ti OR atezolizumab:ab,ti OR tecentriq:ab,ti OR sintilimab:ab,ti OR camrelizumab:ab,ti OR pembrolizumab:ab,ti OR keytruda:ab,ti OR nivolumab:ab,ti OR opdivo:ab,ti OR toripalimab:ab,ti OR tremelimumab:ab,ti OR imjudo:ab,ti OR ipilimumab:ab,ti OR yervoy:ab,ti OR durvalumab:ab,ti OR imfinzi:ab,ti OR penpulimab:ab,ti OR Tislelizumab:ab,ti OR Envafolimab:ab,ti OR Cadonilimab:ab,ti
		6	#4 OR #5
		7	'chemoembolization'/exp OR 'radiotherapy'/exp
		8	((transcatheter:ab,ti OR transarterial:ab,ti) AND chemoemboli*:ab,ti) OR tace:ab,ti OR 'transcatheter arterial embolization':ab,ti OR tae:ab,ti OR

			'hepatic artery infusion chemotherapy':ab,ti OR haic:ab,ti
		9	#7 OR #8
		10	#3 AND #6 AND #9
Cochrane Library	1984 to January 2025	1	MeSH descriptor: [Liver Neoplasms] explode all trees
		2	((hepat*):ti,ab,kw OR (liver):ti,ab,kw) AND ((carcinom*):ti,ab,kw OR (neoplasm*):ti,ab,kw OR (cancer*):ti,ab,kw OR (malign*):ti,ab,kw OR (tumor*):ti,ab,kw OR (tumour*):ti,ab,kw) OR ("HCC"):ti,ab,kw
		3	#1 OR #2
		4	MeSH descriptor: [Immune Checkpoint Inhibitors] explode all trees
		5	("immune checkpoint inhibitor*"):ti,ab,kw OR (ICI):ti,ab,kw OR ("PD 1"):ti,ab,kw OR ("PD LI"):ti,ab,kw OR (CTLA4):ti,ab,kw OR (Atezolizumab):ti,ab,kw OR (Tecentriq):ti,ab,kw OR (Sintilimab):ti,ab,kw OR (Camrelizumab):ti,ab,kw OR (Pembrolizumab):ti,ab,kw OR (Keytrudaw):ti,ab,kw OR (Nivolumab):ti,ab,kw OR (Opdivo):ti,ab,kw OR (Toripalimab):ti,ab,kw OR (Tremelimumab):ti,ab,kw OR (Imjudo):ti,ab,kw OR (Ipilimumab):ti,ab,kw OR (Yervoy):ti,ab,kw OR (Durvalumab):ti,ab,kw OR (Imfinzi):ti,ab,kw OR (Penpulimab):ti,ab,kw OR (Tislelizumab):ti,ab,kw OR (Envafolimab):ti,ab,kw OR (Cadonilimab):ti,ab,kw
		6	#4 OR #5
		7	MeSH descriptor: [Chemoembolization, Therapeutic] explode all trees
		8	(((transcatheter):ti,ab,kw OR (transarterial):ti,ab,kw) AND (chemoemboli*):ti,ab,kw) OR (TACE):ti,ab,kw OR (transcatheter arterial embolization):ti,ab,kw OR (tae):ti,ab,kw OR ("hepatic artery infusion chemotherapy"):ti,ab,kw OR (HAIC):ti,ab,kw
		9	#7 OR #8
		10	#3 AND #6 AND #9
Web of Science(Core Collection)	1900 to January 2025	1	TS=(hepat*) OR TS=(liver)
		2	TS=(carcinom*) OR TS=(neoplasm*) OR TS=(cancer*) OR TS=(malign*) OR TS=(tumor*) OR TS=(tumour*)
		3	#1 AND #2
		4	TS=(HCC)
		5	#3 OR #4
		6	TS=("Immune Checkpoint Inhibitor*") OR TS=(ICI) OR TS=("PD-1") OR TS=("PD-L1") OR TS=(CTLA4) OR TS=(Atezolizumab) OR TS=(Tecentriq) OR TS=(Sintilimab) OR TS=(Camrelizumab) OR TS=(Pembrolizumab) OR TS=(Keytruda) OR TS=(Nivolumab) OR TS=(Opdivo) OR TS=(Toripalimab) OR TS=(Tremelimumab) OR TS=(Imjudo) OR TS=(Ipilimumab) OR TS=(Yervoy) OR TS=(Durvalumab) OR TS=(Imfinzi) OR TS=(Penpulimab) OR TS=(Tislelizumab) OR TS=(Envafolimab) OR TS=(Cadonilimab)
		7	TS=(transcatheter) OR TS=(transarterial)
		8	TS=(chemoemboli*)
		9	#7 AND #8
		10	TS=(TACE) OR TS=("transcatheter arterial embolization") OR TS=(TAE) OR TS=("hepatic artery infusion chemotherapy") OR TS=(HAIC)
		11	#9 OR #10

12	#5 AND #6 AND #11

Table S2 Literature search strategy.

Table S3 Baseline characteristics of studies included in the network meta-analysis

Study Name	Arm	Sample Size	Specific Dosage and Administration Instructions	median Age (Range)	Sex (male%)	Race/Region %	ECOG PS (0, 1, 2)%	Child Pugh score	BCLC Stage	Design
Zuo, 2024	HAIC-TKI-Camre	109	HAIC-mFOLFOX7 regimen; Apatinib: 250mg, PO, QD; Camrelizumab: 200mg, IV, Q3W.	≤ 65(92.7%) > 65(7.3%)	85.30	China	0–1(100%)	NA	B(8.3%), C(91.7%)	Multicenter retrospective cohort study
	TKI-Camre	109	Apatinib: 250mg, PO, QD; Camrelizumab: 200mg, IV, Q3W.	≤ 65(85.3%) > 65(14.7%)	89.00	China	0–1(100%)	NA	B(8.3%), C(91.7%)	
Li, 2024	HAIC-TKI-Camre	83	HAIC-mFOLFOX6 regimen; Camrelizumab: 200 mg IV Q2W (patients<50 kg, 3 mg/kg) ; Rivoceranib: 250 mg, PO, QD.	52.0 (46.0–58.0)	91.60	China	0(98.8%), 1(1.2%)	NA	C(100%)	Multicenter retrospective cohort study
	TKI-Camre	83	Camrelizumab: 200 mg IV Q2W (patients<50 kg, 3 mg/kg) ; Rivoceranib: 250 mg, PO, QD.	52.0 (46.0–58.0)	92.80	China	0(97.6%), 1(2.5%)	NA	C(100%)	
Cao, 2023	TACE-Atez-Bev	61	cTACE regimen; Atezolizumab:1200 mg IV Q3W; Bevacizumab:15 mg/kg IV Q3W.	55.6±11.2	83.60	China	0(49.2%), 1(50.8%)	A(63.9%), B(36.1%)	NA	Bicentric retrospective cohort study
	Atez-Bev	61	Atezolizumab:1200 mg IV Q3W; Bevacizumab:15 mg/kg IV Q3W.	53.9±11.3	83.60	China	0(45.9%), 1(50.9%)	A(68.9%), B(31.1%)	NA	
Zheng, 2024	TACE-Atez-Bev	42	cTACE regimen; Atezolizumab:1200 mg IV Q3W; Bevacizumab:15 mg/kg IV Q3W.	<65(59.5%) ⩾65(40.5%)	85.70	China	NA	A(81.0%), B(19.0%)	NA	Multicenter retrospective cohort study
	TACE	42	Atezolizumab:1200 mg IV Q3W; Bevacizumab:15 mg/kg IV Q3W.	≤ 65(57.1%) > 65(42.9%)	76.20	China	NA	A(85.7%), B(14.3%)	NA	
Zhao, 2023	TACE-Atez-Bev	34	DEB-TACE OR cTACE regimen; Atezolizumab:1200 mg IV Q3W; Bevacizumab:15 mg/kg IV Q3W.	54.5 (41.8–61.3)	84.20	China	0(63.2%), 1(30.6%)	A(100.0%)	B(13.2%), C(80.6%)	Single-center retrospective cohort study
	TACE-TKI	34	DEB-TACE OR cTACE regimen; Lenvatinib:8mg (weight <60 kg) or 12mg (weight ≥60 kg), PO, QD.	55.0 (49.3–66.0)	93.30	China	0(73.3%), 1(26.7%)	A(100.0%)	B(23.3%), C(76.7%)	

Jin, 2023	TACE-TKI-Camre	84	DEB-TACE OR cTACE regimen; Camrelizumab: 200mg, IV, Q3W. Apatinib: 250 mg, PO, QD	57 (49–64)	84.50	China	0(69.0%), 1(31.0%)	A(84.5%), B(15.5%)	B(33.3%), C(66.7%)	Multicenter retrospective cohort study
	TACE	147	DEB-TACE OR cTACE regimen;	57 (51–66)	86.40	China	0(72.1%), 1(27.9%)	A(87.1%), B(12.9%)	B(35.4%), C(64.6%)	
Tang, 2024	TACE-TKI-Camre	70	DEB-TACE OR cTACE regimen; Camrelizumab: 200 mg, IV, Q3W; Lentivanib: 8 mg, PO, QD.	50.0 (45.0–57.0)	95.70	China	0(70.0%), 1(30.0%)	A(88.6%), B(11.4%)	A(18.5%), B(38.6%), C(42.9%)	Single-center retrospective cohort study
	TACE	70	DEB-TACE OR cTACE regimen;	50.00 (45.00–58.00)	91.40	China	0(71.4%), 1(28.6%)	A(84.3%), B(15.7%)	A(21.5%), B(38.5%), C(40.0%)	
Ju, 2021	TACE-TKI-Camre	56	cTACE regimen; Camrelizumab: 200 mg, IV, Q3W; Apatinib: 250 mg, PO, QD.	52 (26–75)	82.10	China	0(48.2%), 1(51.8%)	A(76.8%), B(15.8%)	B(23.2%), C(76.8%)	Single-center retrospective cohort study
	TKI-Camre	52	Camrelizumab: 200 mg, IV, Q3W; Apatinib: 250 mg, PO, QD.	55 (25–79)	84.60	China	0(42.3%), 1(57.7%)	A(84.3%), B(15.9%)	B(9.6%), C(90.4%)	
Guo, 2022	TACE-TKI-Camre	31	cTACE regimen; Sorafenib (400mg bid), Lenvatinib (body weight < 60 kg with 8mg daily, body weight ≥ 60 kg with 12mg daily), or Apatinib (250mg daily); Camrelizumab: 200 mg, IV, Q3W.	＜60(77.4%) ⩾60(22.6%)	83.90	China	0–1(71.0%), 2(29.0%)	A(67.7%), B(32.3%)	A(6.5%), B(16.1%), C(77.4%)	Single-center retrospective cohort study
	TKI-Camre	32	Sorafenib (400mg bid), Lenvatinib (body weight < 60 kg with 8mg daily, body weight ≥ 60 kg with 12mg daily), or Apatinib (250mg daily); Camrelizumab: 200 mg, IV, Q3W.	＜60(52.2%) ⩾60(47.8%)	95.70	China	0–1(65.2%), 2(34.8%)	A(60.9%), B(39.1%)	A(4.3%), B(13.1%), C(82.6%)	
Chen, 2021	TACE-TKI-Pembro	70	cTACE regimen; Pembrolizumab: 200mg, IV, Q3W; Lentivanib: 8 mg, PO, QD.	＜50(45.7%) ⩾50(54.3%)	52.90	China	0(38.6%), 1(61.4%)	NA	B(67.1%), C(32.9%)	Multicenter retrospective cohort study

	TACE-TKI	72	cTACE regimen<br>Lentivanib: 9 mg, PO, QD.	＜50(50.0%)<br>⩾50(50.0%)	52.80	China	0(41.7%),<br>1(58.3%)	NA	B(62.5%),<br>C(37.5%)	
Xiang, 2023	TACE-TKI-Camre	33	DEB-TACE OR cTACE regimen;<br>Lenvatinib: body weight (≥60 kg, 12 mg or <60 kg, 8 mg), QD.<br>Camrelizumab: 3mg/kg, IV, Q3W;	51.0 ± 12.2	84.80	China	0(66.7%),<br>1(33.3%)	A(75.8%),<br>B(24.2%)	B(30.3%),<br>C(69.7%)	Single-center retrospective cohort study
	TACE-TKI	49	DEB-TACE OR cTACE regimen;<br>Lenvatinib: body weight (≥60 kg, 12 mg or <60 kg, 8 mg), QD.	51.7 ± 11.2	91.80	China	0(77.6%),<br>1(22.4%)	A(83.7%),<br>B(16.3%)	B(44.9%),<br>C(55.1%)	
Sun, 2022	TACE-TKI-Camre	39	cTACE regimen;<br>Lenvatinib: body weight (≥60 kg, 12 mg or <60 kg, 8 mg), PO, QD;<br>Camrelizumab: 200 mg, IV, Q3W.	54.84 ± 9.249	80.60	China	0(61.3%),<br>1(38.7%)	A(77.4%),<br>B(22.6%)	B(35.5%),<br>C(64.5%)	Bicentric retrospective cohort study
		70	cTACE regimen;<br>Lenvatinib: body weight (≥60 kg, 12 mg or <60 kg, 8 mg), PO, QD;	51,77 ± 9,791	88.50	China	0(42.3%),<br>1(57.7%)	A(82.7%),<br>B(17.3%)	B(32.7%),<br>C(67.3%)	
Lu, 2023	TACE-TKI-Tori	81	cTACE regimen;<br>Donafenib: 200 mg, PO, BID;<br>Toripalimab: 240 mg, IV, Q3W.	51.9±12.4	80.20	China	0(38.3%),<br>1(43.2%),<br>2(18.5%)	NA	B(27.2%),<br>C(72.8%)	Single-center retrospective cohort study
	TACE-TKI	88	cTACE regimen;<br>Sorafenib: 400 mg, PO, BID.	53.9±12.1	76.10	China	0(37.5%),<br>1(46.6%),<br>2(15.9%)	NA	B(31.8%),<br>C(68.2%)	
Zhu, 2022	TACE-TKI-Camre	34	cTACE regimen;<br>Apatinib: 250mg, PO, QD;<br>Camrelizumab: 200mg, IV, Q3W.	＜60(67.6%)<br>⩾60(32.4%)	85.30	China	0(55.9%),<br>1(44.1%)	A(88.2%),<br>B(11.8%)	B(38.2%),<br>C(61.8%)	Bicentric retrospective cohort study
	TACE-TKI	68	cTACE regimen;<br>Apatinib: 250mg, PO, QD.	＜60(60.3%)<br>⩾60(39.7%)	85.30	China	0(50.0%),<br>1(50.0%)	A(82.4%),<br>B(17.6%)	B(38.2%),<br>C(61.8%)	
Duan, 2023	TACE-TKI-Camre	449	cTACE regimen;<br>Apatinib: 250mg, PO, QD;<br>Camrelizumab: 200mg, IV, Q3W.	52.7 ± 8.9	82.90	China	0(58.4%),<br>1(57.0%)	A(39.0%),<br>B(61.0%)	B(17.4%),<br>C(82.6%)	Multicenter retrospective cohort study
	TACE-TKI	449	cTACE regimen;	52.7 ± 9.1	81.70	China	0(57.0%),	A(41.2%),		

			Apatinib: 250mg, PO, QD.				1(43.0%)	B(58.8%)	B(16.7%), C(83.3%)	
Liu, 2023(1)	TACE-TKI-Camre	37	cTACE regimen; Apatinib: 250mg, PO, QD; Camrelizumab: 200mg, IV, Q3W.	＜60(67.6%) ≥60(32.4%)	86.50	China	0(73.0%), 1(27.0%)	A(86.5%), B(13.5%)	B(51.4%), C(48.6%)	Single-center retrospective cohort study
	TACE-TKI	39	cTACE regimen; Apatinib: 250mg, PO, QD.	＜60(46.2%) ≥60(53.8%)	92.30	China	0(61.5%), 1(38.5%)	A(89.7%), B(10.3%)	B(61.5%), C(38.5%)	
Jiang, 2024(1)	TACE-TKI-Camre	44	cTACE regimen; Sorafenib 400mg, P.O BID or lenvatinib (<60kg, 8mg/day; ≥60kg, 12mg/day); Camrelizumab: 200mg, IV, Q3W.	≥65(13.64%)	84.09	China	NA	A(70.45%), B(29.55%)	B（18.18%), C(81.82%)	Single-center retrospective cohort study
	TACE-TKI	83	cTACE regimen; Sorafenib 400mg, P.O BID or lenvatinib (<60kg, 8mg/day; ≥60kg, 12mg/day).	≥65(22.89%)	83.13	China	NA	A(80.72%), B(19.28%)	B（13.25%), C(86.75%)	
Qu, 2022	TACE-TKI-Tori	30	cTACE regimen; Lenvatinib :8mg P.O QD; Toripalimab： 240mg ivdrip Q3W	55.5 (47.8- 64.3)	86.70	China	NA	A(93.3%), B(6.7%)	B（3.3%), C(96.7%)	
	TACE-TKI	21	cTACE regimen; Lenvatinib :8mg P.O QD.	50.0 (45.0-61.0)	95.20	China	NA	A(100%), B(0%)	B（14.3%), C(85.7%)	
Jiang, 2024(2)	TACE-TKI-Tisle	68	cTACE regimen; Lenvatinib: body weight (≥60 kg, 12 mg or <60 kg, 8 mg), PO, QD; Tislelizumab：200mg, IV, Q3W.	＜60(75.0%) ≥60(25.0%)	86.80	China	0(79.4%), 1(20.6%)	A(89.7%), B(10.3%)	B(33.8%), C(66.2%)	Single-center retrospective cohort study
	TACE-TKI	68	cTACE regimen; Lenvatinib: body weight (≥60 kg, 12 mg or <60 kg, 8 mg), PO, QD.	＜60(63.2%) ≥60(36.8%)	13.20	China	0(77.9%), 1(22.1%)	A(86.8%), B(13.2%)	B(32.4%), C(67.6%)	

Zhao, 2024	TACE-TKI-Tisle	103	cTACE regimen;<br>Lenvatinib: body weight (≥60 kg, 12 mg or <60 kg, 8 mg), PO, QD;<br>Tislelizumab：200mg, IV, Q3W.	≤70(87.4%)<br>＞70(12.6%)	75.70	China	NA	A(82.57%),<br>B(17.43%)	B(16.5%),<br>C(83.5%)	Multicenter retrospective cohort study
	TACE-TKI	66	cTACE regimen;<br>Lenvatinib: body weight (≥60 kg, 12 mg or <60 kg, 8 mg), PO, QD.	≤70(81.8%)<br>＞70(18.2%)	89.40	China	NA	A(83.82%),<br>B(16.18%)	B(16.7%),<br>C(83.3%)	
Wu, 2025	TACE-TKI-Sinti	15	cTACE regimen;<br>Lenvatinib: body weight (≥60 kg, 12 mg or <60 kg, 8 mg), PO, QD;<br>Sintilimab：200mg, Q3W, ivdrip.	< 60(46.7%)<br>≥60(53.3%)	0.93	China	0(40%)<br>1（60%）	A(100%),<br>B(0%)	B(73.3%),<br>C(26.7%)	Single-center retrospective cohort study
	TACE-TKI	15	cTACE regimen;<br>Lenvatinib: body weight (≥60 kg, 12 mg or <60 kg, 8 mg), PO, QD;	< 60(46.7%)<br>≥60(53.3%)	86.70	China	0(20%)<br>1（80%）	A(93.3%),<br>B(6.7%)	B(73.3%),<br>C(26.7%)	
Lang, 2023	TACE-TKI-Sinti	75	cTACE regimen;<br>Lenvatinib: body weight (8 mg), PO, QD;<br>Sintilimab：200mg, Q3W, ivdrip	≤65 (76.0%)<br>>65 (24.0%)	88.00	China	0(64.0%)<br>1(32.0%)<br>2 (4.0%)	A(78.7%),<br>B(21.3%)	B(42.7%),<br>C(57.3%)	Single-center retrospective cohort study
	TKI-Sinti	39	Lenvatinib: body weight (8 mg), PO, QD;<br>Sintilimab：200mg, Q3W, ivdrip	≤65 (74.4%)<br>>65 (25.6%)	87.20	China	0(71.8%)<br>1(20.5%)<br>2 (7.7%)	A(76.9%),<br>B(23.1%)	B(35.9%),<br>C(64.1%)	
Chen, 2024(1)	TACE-TKI-Tisle	66	cTACE regimen;<br>Lenvatinib: body weight (≥60 kg, 12 mg or <60 kg, 8 mg), PO, QD;<br>Tislelizumab：200mg, IV, Q3W.	55.8 ± 11.2	92.40	China	NA	A(89.4%),<br>B(10.6%)	NA	Multicenter retrospective cohort study
	TACE-TKI	45	cTACE regimen;<br>Lenvatinib: body weight (≥60 kg, 12 mg or <60 kg, 8 mg), PO, QD;	56.6 ± 12.1	93.30	China	NA	A(88.9%),<br>B(11.1%)	NA	
	TACE	38	cTACE regimen;	56.5 ± 13.0	92.10	China	NA	A(84.2%),<br>B(15.8%)	NA	
Wu, 2023(1)	TACE-TKI-Camre	57	DEB-TACE OR cTACE regimen;<br>Lenvatinib: body weight (≥60 kg, 12 mg or <60 kg, 8 mg), PO, QD;	53.18±9.25	86.00	China	1(70.2%),<br>2(29.8%)	A(91.2%),<br>B(8.8%)	NA	Single-center retrospective cohort study

			Camrelizumab: 3mg/kg, IV, Q3WLenvatinib: body weight (≥60 kg, 12 mg or <60 kg, 8 mg), PO, QD.							
	TACE-Camre	41	DEB-TACE OR cTACE regimen; Camrelizumab: 3mg/kg, IV, Q3WLenvatinib.	55.27±10.48	82.90	China	1(58.8%), 2(12.2%)	A(87.8%), B(12.2%)	NA	
	TACE	43	DEB-TACE OR cTACE regimen;	53.5±9.35	74.40	China	1(90.7%), 2(9.3%)	A(90.7%), B(9.3%)	NA	
Sangro,2025	TACE-Bev-Durva	204	DEB-TACE OR cTACE regimen; Durvalumab: 1120 mg, IV, Q3W Bevacizumab: 15 mg/kg, IV, Q3W	64.5 (58.0-73.0)	79.00	Asia (62.0%), White (27.0%), American Indian or Alaska Native (6.0%), Black or African American (1.0%), Other (4.0%)	0 (82.0%), 1 (18.0%)	A (98.0%), B (2.0%)	A (25.0%), B (57.0%), C (17.0%)	Phase 3 Randomized Controlled Trial
	TACE-Durva	207	DEB-TACE OR cTACE regimen; durvalumab: 1120 mg, IV, Q3W	65.0 (59.0-72.0)	75.00	Asia (60.0%),White (30.0%), American Indian or Alaska Native (2.0%), Black or African American (1.0%), Other (6.0%)	0 (84.0%), 1 (16.0%)	A (97.0%), B (3.0%)	A (29.0%), B (55.0%), C (16.0%)	
	TACE	205	DEB-TACE OR cTACE regimen;	66.0 (59.0-71.0)	80.00	Asia (61.0%),White (29.0%), American Indian or Alaska Native (2.0%), Black or African American (1.0%), Other (6.0%)	0 (85.0%), 1 (15.0%)	A (98.0%), B (2.0%)	A (24.0%), B (60.0%), C (15.0%)	
Kudo,2025	TACE-TKI-Pembro	237	DEB-TACE OR cTACE regimen; Lenvatinib: body weight (≥60 kg, 12 mg or <60 kg, 8 mg), PO, QD; Pembrolizumab: 400 mg, IV, Q6W.	65 (57-72)	81.00	Asian (71.0%), White (22.0%), Black or African American (1.0%), American Indian or Alaska Native (1.0%), Other (6.0%)	0 (91.0%), 1 (9.0%)	A (86.0%), B (14.0%)	A (34.0%), B (57.0%), C (9.0%)	Phase 3 Randomized Controlled Trial

	TACE	205	DEB-TACE OR cTACE regimen;	66 (59-73)	85.00	Asian (74.0%), White (19.0%), Black or African American (1.0%), American Indian or Alaska Native (1.0%), Other (5.0%)	0 (88.0%), 1 (12.0%)	A (89.0%), B (11.0%)	A (28.0%), B (60.0%), C (12.0%)	
Li, 2025	TACE-Bev-Ate	52	DEB-TACE OR cTACE regimen; Atezolizumab:1200 mg, IV, q3w; Bevacizumab:15 mg/kg, IV, q3w.	52.6±11.6	86.50	China	0(28.8%), 1(71.2%)	A(76.9%), B(23.1%)	NA	Single-center retrospective cohort study
	Bev-Ate	31	Atezolizumab:1200 mg, IV, q3w; Bevacizumab:15 mg/kg, IV, q3w.	53.6 ± 13.5	80.60	China	0(41.9%), 1(58.1%)	A(61.2%), B(38.7%)	NA	
Cai, 2025	TAE-HAIC-Len-Tils e	153	TAE-HAIC regimen; Lenvatinib: body weight (≥60 kg, 12 mg or <60 kg, 8 mg), PO, QD; Tislelizumab: 200mg, IV, Q3W.	56.7 ±10.5	94.80	China	0(90.2%), 1(9.8%)	A75.8%), B(24.2%)	B(35.3%), C(64.7%)	Multicenter retrospective cohort study
	TAE−HAIC−Bev−A tez	51	TAE-HAIC regimen; atezolizumab:1200 mg, IV, Q3W. evacizumab :15 mg/kg, IV, Q3W.	57.0 ± 12.3	92.20	China	0(92.2%), 1(7.8%)	A(80.0%), B(20.0%)	B(35.3%), C(64.7%)	
Chen, 2023	TAE-HAIC-TKI-Tisl e	50	TAE-HAIC regimen; Lenvatinib: body weight(≥60 kg, 12 mg or <60 kg, 8 mg), PO, QD; Tislelizumab: 200mg, IV, Q3W.	56 (43–62)	84.00	China	0(80.0%), 1(20.0%)	A(72.0%), B(28.0%)	NA	Multicenter retrospective cohort study
	HAIC-TKI-Tisle	50	HAIC regimen; Lenvatinib: body weight (≥60 kg, 12 mg or <60 kg, 8 mg), PO, QD; Tislelizumab: 200mg, IV, Q3W.	55 (36–71)	92.00	China	0(76.0%), 1(24.0%)	A(70.0%), B(30.0%)	NA	
Zhao, 2025	HAIC-TKI-Tisle	47	HAIC-FOLFOX6 regimen; Lenvatinib: body weight (≥60 kg, 12 mg or <60 kg, 8 mg), PO, QD; Tislelizumab: 200mg, IV, Q3W.	⩾50(53.2%) <50（46.8%）	87.20	China	0(95.7%), 1(4.3%)	A(93.6%), B(6.4%)	NA	Multicenter retrospective cohort study

Li, 2023(1)	TACE-TKI-Camre	69	cTACE regimen; Lenvatinib: body weight (≥60 kg, 12 mg or <60 kg, 8 mg), PO, QD; Camrelizumab: 200 mg, IV, Q3W.	49.0-64.0	78.30	China	0(72.5%) 1(27.5%)	A(60.9%), B(39.1%)	NA	Multicentre,single-arm, prospective study
Wu, 2023(2)	TACE-TKI-Camre	55	cTACE regimen; Lenvatinib: body weight (≥60 kg, 12 mg or <60 kg, 8 mg), PO, QD; Camrelizumab: 200 mg, IV, Q3W.	46.0–62.0	81.80	China	0(85.5%) 1(14.5%)	A(100%), B(0%)	B(21.8%), C(78.2%)	Multicentre,single-arm, prospective study
Li, 2023(2)	TACE-TKI-Camre	87	cTACE regimen; Sorafenib:400 mg, P.O, BID/regorafenib:80-160 mg, once daily, for 21 days, 7 days off/Lenvatinib: body weight (≥60 kg, 12 mg or <60 kg, 8 mg), PO, QD; Camrelizumab: 200 mg, IV, Q3W.	＜65 (80.5%) ≥65 (19.5%)	93.10	China	0(52.9%) 1(32.2%) NE（14.9%）	A(58.6%), B(33.3%), NE（8.1%）	NA	Multicentre,single-arm, prospective study
Cai, 2023	TACE-TKI-Sinti	30	cTACE regimen; Lenvatinib: body weight (≥60 kg, 12 mg or <60 kg, 8 mg), PO, QD; Sintilimab：200mg, Q3W, ivdrip	49.4 ± 9.9	86.70	China	0(86.7%) 1(13.3%)	A(96.7%), B(3.3%),	C(100%)	Single-center ,single-arm, prospective study
Chen, 2024(2)	TACE-TKI-Enva	38	cTACE regimen; Lenvatinib: body weight (≥60 kg, 12 mg or <60 kg, 8 mg), PO, QD; Envafolimab (300mg, subcutaneous injection , Q3W)	28–73	81.60	China	0(100%)	A(100%),	B(44.7%), C(55.3%)	Single-center ,single-arm, prospective study
Zhang, 2023	HAIC-TKI-Camre	35	HAIC-FOLFOX regimen; Lenvatinib: body weight (≥60 kg, 12 mg or <60 kg, 8 mg), PO, QD; Camrelizumab: 200 mg, IV, Q3W.	⩾50(42.9%) <50（57.1%）	91.40	China	0(45.7%) 1(51.4%) 2 (2.9%)	A(100%), B(0%)	C(100%)	Single-center ,single-arm, prospective study
He, 2023	HAIC-TKI-Tori	30	HAIC-FOLFOX regimen; Lenvatinib: body weight (≥60 kg, 12 mg or <60 kg, 8 mg), PO, QD; Toripalimab: 240 mg, IV, Q3W.	38-52.5	90.00	China	0(30.0%) 1(70.0%)	A(100%), B(0%)	C(100%)	Single-center ,single-arm, prospective study

Lai, 2022	HAIC-TKI-Tori	36	HAIC-FOLFOX regimen;<br>Lenvatinib: body weight (≥60 kg, 12 mg or <60 kg, 8 mg), PO, QD;<br>Toripalimab: 240 mg, IV, Q3W.	39-57	91.70	China	0(45.7%)<br>1(51.4%)<br>2 (2.9%)	A(100%),<br>B(0%)	NA	Single-center ,single-arm, prospective study
Tai, 2021	TARE-Nivo	36	TARE(SIR-Spheres Y90 resin microspheres,<br>Sirtex, Singapore) regimen;<br>Nivolumab:240 mg, IV, Q3W	59.7-70.9	78.00	Chinese (69%)<br>Indian (6%)<br>Other (25%)	0(72.0%)<br>1(25.0%)<br>2 (3.0%)	A(100%),<br>B(0%)	A(3.0%),<br>B(31.0%),<br>C(66.0%)	Single-center ,single-arm, prospective study
Yu, 2024	TARE-Pembro	29	TARE regimen;<br>Nivolumab:200 mg, IV, Q3W	33-79	89.00	Caucasian（85%）<br>African American （4%）<br>American Indian or Alaska Native（4%）<br>Unknown（7%）	0(48.0%)<br>1(52.0%)	A(96.0%),<br>B(4.0%)	NA	Multicentre,single-arm, prospective study
Liu, 2023(2)	HAIC-IBI305-Sinti	29	FOLFOX-HAIC regimen;<br>IBI305:7.5 mg/kg, IV, Q3W<br>Sintilimab:200 mg, IV, Q3W	47.5–64.0	72.20	China	0(96.6%)<br>1(3.4%)	A(96.6%),<br>B(3.4%)	B(13.8%),<br>C(86.2%)	Single-center ,single-arm, prospective study
Ren, 2024	TACE-TKI-Pembro-chemo	40	cTACE regimen;<br>Lenvatinib: body weight (≥60 kg, 12 mg or <60 kg, 8 mg), PO, QD;<br>Pembrolizumab:200mg, IV, Q3W<br>cyclophosphamide, 50mg, P.O, QD	54.2±10.6	82.40	China	NA	A(85.3%),<br>B(14.7%)	A-B(20.6%)<br>C(79.4%)	Single-center ,single-arm, prospective study
Mu, 2025	TACE-Sinti-Bev	34	TACE regimen;<br>Sintilimab: 200 mg IA ;<br>Bevacizumab: 7.5mg/kg, IA.	53.0 (45.0–59.0)	97.10	China	NA	A(88.2%),<br>B(11.8%)	NA	single-center, single-arm, prospective study
Gao, 2025	HAIC-TKI-Sinti	36	HAIC-FOLFOX6 regimen;<br>Donafenib: 200 mg, PO, BID ;<br>Sintilimab: 200 mg, Q3W.	58.8(9.3)	83.30	China	0(97.2%),<br>1(2.8%)	NA	A(11.1%),<br>B(33.3%),<br>C(55.6%)	single-center, single-arm, prospective study
Shen, 2025	HAIC-TKI-Camre	25	HAIC-FOLFOX6 regimen;<br>Sorafenib: 400 mg, PO, BID ;<br>Camrelizumab: 200 mg, IA.	48.0 (34.0–67.0)	96.00	China	0(76%),<br>1(24%)	A(88.0%),<br>B(12.0%)	NA	single-center, single-arm, prospective study

TACE=transcatheter arterial chemoembolization. c-TACE=conventional TACE. DEB-TACE=drug eluting beads TACE. HAIC=hepatic arterial infusion chemotherapy. TARE=transarterial radioembolization. FOLFOX=folinic acid and oxaliplatin with 5-fluorouracil. ECOG PS=eastern cooperative oncology group performance status score.BCLC = Barcelona Clinic Liver Cancer staging system. NA=not applicable.

Table S4 Risk of bias assessment for the included cohort studies according to the NOS (Newcastle-Ottawa Scale) evaluation criteria

Study	Selection				Comparability	Outcome			Total scores
	Represent ativeness of the exposed cohort	Selection of the nonexposed cohort	Ascertainment of exposure	Demonstration that outcome of interest Was not present at start of study	Comparability of cohorts on the basis of the design or analysis	Assessment of outcome	Was follow-up long enough for outcomes to occur	Adequacy of follow-up of cohorts	
Zuo, 2024	*	*	*	*	**	*	*	*	9
Li, 2024		*	*	*	**	*		*	7
Cao, 2023	*	*	*	*	**	*		*	8
Zheng, 2024		*	*	*	**	*		*	7
Zhao, 2023	*	*	*	*	**	*		*	8
Jin, 2023	*	*	*	*	**	*		*	8
Tang, 2024	*	*	*	*	**	*		*	8
Ju, 2021	*	*	*	*	*	*		*	7
Guo, 2022		*	*	*	*	*			5
Chen, 2021	*	*	*	*	*	*	*		7
Xiang, 2023		*	*	*	*	*	*	*	7
Sun, 2022	*	*	*	*	*	*	*		7
Lu, 2023		*	*	*	*	*			5
Zhu, 2022	*	*	*	*	**	*	*	*	9
Duan, 2023	*	*	*	*	**	*	*	*	9
Liu, 2023		*	*	*	*	*			5
Jiang, 2024(1)	*	*	*	*	*	*		*	7
Qu, 2022	*	*	*	*	*	*			6
Jiang, 2024(2)	*	*	*	*	**	*		*	8
Zhao, 2024	*	*	*	*	*	*	*		7
Wu， 2025		*	*	*	**	*			6
Lang,2023	*	*	*	*	**	*			7
Chen, 2024(1)	*	*	*	*	*	*	*		7
Wu, 2023(1)		*	*	*	*	*			5
Li, 2025	*	*	*	*	*	*	*		7
Chen, 2023	*	*	*	*	*	*	*		7
Cai, 2025	*	*	*	*	**	*	*		8
Zhao, 2025	*	*	*	*	**	*	*		8

Table S5 Bayesian ranking results of network meta-analysis

PFS	Rank Probability (%)														
	Rank 1st	Rank 2st	Rank 3st	Rank 4st	Rank 5st	Rank 6st	Rank 7st	Rank 8st	Rank 9st	Rank 10st	Rank 11st	Rank 12st	Rank 13st	Rank 14st	Rank 15st
Bev-Atez	0.01	0.01	0.02	0.02	0.03	0.04	0.06	0.07	0.08	0.08	0.09	0.12	0.13	0.14	0.10
HAIC-TKI-Camre	0.05	0.04	0.04	0.04	0.05	0.06	0.06	0.06	0.06	0.06	0.07	0.09	0.15	0.16	0.02
TACE	0.00	0.00	0.00	0.00	0.00	0.01	0.02	0.05	0.10	0.16	0.21	0.21	0.15	0.07	0.02
TACE-Bev-Atez	0.01	0.03	0.05	0.07	0.09	0.12	0.13	0.12	0.10	0.08	0.07	0.06	0.04	0.02	0.00
TACE-Camre	0.09	0.10	0.11	0.11	0.11	0.11	0.09	0.07	0.06	0.04	0.04	0.03	0.02	0.01	0.01
TACE-Durva	0.01	0.01	0.02	0.03	0.04	0.05	0.07	0.08	0.09	0.09	0.10	0.12	0.12	0.10	0.07
TACE-Bev-Durva	0.02	0.03	0.04	0.05	0.07	0.09	0.10	0.10	0.10	0.09	0.09	0.08	0.07	0.05	0.03
TACE-TKI	0.00	0.00	0.00	0.00	0.01	0.03	0.09	0.15	0.19	0.19	0.16	0.10	0.05	0.02	0.00
TACE-TKI-Camre	0.01	0.08	0.20	0.26	0.21	0.13	0.07	0.03	0.01	0.00	0.00	0.00	0.00	0.00	0.00
TACE-TKI-Pembro	0.03	0.06	0.09	0.11	0.14	0.14	0.13	0.10	0.07	0.05	0.03	0.02	0.01	0.01	0.00
TACE-TKI-Sinti	0.09	0.11	0.12	0.11	0.10	0.10	0.09	0.07	0.05	0.04	0.04	0.03	0.02	0.02	0.00
TACE-TKI-Tisle	0.32	0.31	0.17	0.09	0.05	0.03	0.02	0.01	0.00	0.00	0.00	0.00	0.00	0.00	0.00
TACE-TKI-Tori	0.35	0.19	0.12	0.08	0.07	0.05	0.04	0.03	0.02	0.01	0.01	0.01	0.01	0.00	0.00
TKI-Camre	0.00	0.00	0.01	0.01	0.01	0.02	0.02	0.03	0.03	0.04	0.05	0.06	0.11	0.25	0.37
TKI-Sinti	0.01	0.01	0.01	0.02	0.02	0.02	0.03	0.03	0.04	0.04	0.05	0.06	0.12	0.16	0.39

OS	Rank Probability (%)												
	Rank 1st	Rank 2st	Rank 3st	Rank 4st	Rank 5st	Rank 6st	Rank 7st	Rank 8st	Rank 9st	Rank 10st	Rank 11st	Rank 12st	Rank 13st
Bev-Atez	0.00	0.01	0.02	0.02	0.03	0.04	0.07	0.09	0.10	0.12	0.14	0.17	0.20
HAIC-TKI-Camre	0.02	0.09	0.08	0.07	0.08	0.10	0.11	0.11	0.10	0.09	0.08	0.07	0.00
TACE	0.00	0.00	0.00	0.00	0.00	0.00	0.01	0.03	0.08	0.19	0.31	0.27	0.11
TACE-Bev-Atez	0.01	0.10	0.11	0.10	0.12	0.14	0.13	0.10	0.08	0.05	0.03	0.01	0.00
TACE-Camre	0.08	0.33	0.16	0.11	0.09	0.08	0.06	0.04	0.02	0.01	0.01	0.00	0.00
TACE-TKI	0.00	0.00	0.00	0.00	0.00	0.02	0.09	0.21	0.30	0.24	0.11	0.03	0.00
TACE-TKI-Camre	0.00	0.06	0.20	0.27	0.24	0.14	0.06	0.02	0.00	0.00	0.00	0.00	0.00
TACE-TKI-Pembro	0.00	0.03	0.05	0.07	0.11	0.16	0.18	0.16	0.11	0.07	0.04	0.02	0.00
TACE-TKI-Sinti	0.02	0.11	0.12	0.11	0.12	0.13	0.13	0.10	0.07	0.05	0.03	0.02	0.00
TACE-TKI-Tisle	0.01	0.16	0.22	0.20	0.16	0.12	0.07	0.04	0.01	0.00	0.00	0.00	0.00

TACE-TKI-Tori	0.85	0.10	0.03	0.01	0.01	0.00	0.00	0.00	0.00	0.00	0.00	0.00	0.00
TKI-Camre	0.00	0.00	0.00	0.00	0.00	0.01	0.02	0.03	0.05	0.08	0.13	0.25	0.42
TKI-Sinti	0.01	0.02	0.03	0.03	0.03	0.04	0.06	0.08	0.08	0.10	0.11	0.16	0.26

	Rank Probability (%)														
ORR	Rank 1st	Rank 2st	Rank 3st	Rank 4st	Rank 5st	Rank 6st	Rank 7st	Rank 8st	Rank 9st	Rank 10st	Rank 11st	Rank 12st	Rank 13st	Rank 14st	Rank 15st
Bev-Atez	0.00	0.00	0.00	0.00	0.00	0.00	0.00	0.00	0.00	0.00	0.00	0.01	0.02	0.04	0.91
HAIC-TKI-Camre	0.01	0.01	0.02	0.02	0.03	0.03	0.04	0.06	0.08	0.10	0.12	0.16	0.24	0.08	0.01
TACE	0.00	0.00	0.00	0.00	0.00	0.00	0.00	0.01	0.04	0.13	0.25	0.29	0.19	0.09	0.00
TACE-Bev-Atez	0.02	0.05	0.08	0.11	0.12	0.11	0.12	0.12	0.11	0.08	0.05	0.03	0.01	0.00	0.00
TACE-Camre	0.01	0.02	0.03	0.04	0.05	0.06	0.07	0.09	0.12	0.13	0.13	0.11	0.08	0.06	0.00
TACE-Durva	0.02	0.03	0.04	0.05	0.06	0.06	0.07	0.10	0.13	0.14	0.12	0.09	0.06	0.05	0.00
TACE-Bev-Durva	0.03	0.03	0.05	0.06	0.07	0.07	0.08	0.10	0.13	0.13	0.10	0.07	0.05	0.03	0.00
TACE-TKI	0.00	0.00	0.01	0.03	0.09	0.17	0.22	0.21	0.15	0.08	0.03	0.01	0.00	0.00	0.00
TACE-TKI-Camre	0.00	0.02	0.07	0.16	0.22	0.21	0.16	0.10	0.04	0.02	0.00	0.00	0.00	0.00	0.00
TACE-TKI-Pembro	0.07	0.11	0.16	0.16	0.13	0.10	0.08	0.07	0.05	0.03	0.02	0.01	0.00	0.00	0.00
TACE-TKI-Sinti	0.21	0.15	0.14	0.10	0.07	0.06	0.06	0.06	0.05	0.04	0.03	0.02	0.01	0.00	0.00
TACE-TKI-Tisle	0.29	0.30	0.20	0.11	0.05	0.03	0.01	0.01	0.00	0.00	0.00	0.00	0.00	0.00	0.00
TACE-TKI-Tori	0.32	0.24	0.17	0.10	0.06	0.04	0.03	0.02	0.01	0.01	0.00	0.00	0.00	0.00	0.00
TKI-Camre	0.00	0.00	0.00	0.00	0.00	0.00	0.01	0.01	0.02	0.04	0.07	0.11	0.24	0.44	0.04
TKI-Sinti	0.03	0.05	0.04	0.05	0.04	0.04	0.04	0.05	0.07	0.08	0.08	0.10	0.09	0.21	0.03

	Rank Probability (%)							
Safty	Rank 1st	Rank 2st	Rank 3st	Rank 4st	Rank 5st	Rank 6st	Rank 7st	Rank 8st
TACE	0.00	0.02	0.05	0.12	0.21	0.24	0.22	0.13
TACE-Camre	0.20	0.09	0.07	0.07	0.06	0.08	0.06	0.37
TACE-Durva	0.04	0.08	0.11	0.17	0.18	0.17	0.15	0.10
TACE-Bev-Durva	0.12	0.19	0.21	0.17	0.14	0.09	0.05	0.03
TACE-TKI	0.01	0.05	0.10	0.12	0.12	0.16	0.25	0.19
TACE-TKI-Camre	0.24	0.29	0.21	0.13	0.08	0.04	0.01	0.00
TACE-TKI-Pembro	0.31	0.19	0.13	0.11	0.09	0.07	0.05	0.04

TACE-TKI-Tisle	0.08	0.10	0.11	0.11	0.11	0.15	0.20	0.14

OS=overall survival. PFS=progression-free survival. ORR=objective response rate. TACE=transcatheter arterial chemoembolization. HAIC=hepatic arterial infusion chemotherapy. TKI=tyrosine-kinase inhibitor. Bev=bevacizumab. Camre=camrelizumab. Pembro=pembrolizumab. Tisle=tislelizumab. Tori=toripalimab. Atez=atezolizumab. Sinti=sintilimab. Durva=Durvalumab.

Table S6 Comparison of the fit goodness between consistency and inconsistency models based on DIC values in network meta-analysis.

Outcome	Model	DIC
Progression-free survival	Consistency	54.266
	Inconsistency	55.059
Overall survival	Consistency	48.367
	Inconsistency	50.259
Objective response rate	Consistency	103.951
	Inconsistency	106.505
Grade ≥ 3 adverse events	Consistency	33.884
	Inconsistency	33.761

The DIC provides a measure of model fit adjusted with the complexity of the model, with lower values correspond to preferable models and differences of 5 considered important.[https://rss.onlinelibrary.wiley.com/doi/10.1111/1467-9868.00353]DIC, Deviance information criterion.

Table S7 Inconsistency analysis of network meta-analysis results

Outcome	Comparisons	Pooled Mean Difference (95% CI)		
		Pooled Pairwise	Pooled Network	I^2 (%)
PFS				
	TACE-Bev-Atez vs Bev-Atez	-0.37 (-1.10 , 0.34)	-0.37 (-1.10 , 0.29)	93.60%
	TKI-Camre vs HAIC-TKI-Camre	0.55 (-0.17 , 1.30)	0.55 (-0.11 , 1.20)	0.00%
	TACE-Bev-Atez vs TACE	-0.75 (-1.90 , 0.39)	-0.37 (-1.10 , 0.42)	51.60%
	TACE-Camre vs TACE	-0.53 (-1.70 , 0.62)	-0.61 (-1.5 , 0.28)	-
	TACE-Durva vs TACE	-0.06 (-1.10 , 0.95)	-0.06 (-0.97 , 0.85)	-
	TACE-Bev-Durva vs TACE	-0.26 (-1.30 , 0.76)	-0.26 (-1.2 , 0.65)	-
	TACE-TKI vs TACE	-0.63 (-1.80 , 0.48)	-0.15 (-0.64 , 0.32)	67.80%
	TACE-TKI-Camre vs TACE	-0.60 (-1.20 , -0.00)	-0.71 (-1.20 , -0.28)	0.00%
	TACE-TKI-Pembro vs TACE	-0.42 (-1.40 , 0.60)	-0.53 (-1.20 , 0.17)	0.00%
	TACE-TKI-Tisle vs TACE	-0.87 (-2.1 , 0.33)	-1.0 (-1.7 , -0.39)	0.00%
	TACE-TKI vs TACE-Bev-Atez	-0.19 (-1.3 , 0.96)	0.21 (-0.57.0.99)	53.40%
	TACE-TKI-Camre vs TACE-Camre	-0.014 (-1.2 , 1.1)	-0.10 (-1.0 , 0.80)	-
	TACE-Bev-Durva vs TACE-Durva	-0.20 (-1.20 , 0.82)	-0.20 (-1.10 , 0.70)	-
	TACE-TKI-Camre vs TACE-TKI	-0.63 (-1.1.-0.20)	-0.56 (-0.93 , -0.20)	83.10%
	TACE-TKI-Pembro vs TACE-TKI	-0.51 (-1.60 , 0.55)	-0.38 (-1.10 , 0.34)	0.00%
	TACE-TKI-Sint vs TACE-TKI	-0.46 (-1.50 , 0.58)	-0.46 (-1.40 , 0.49)	-
	TACE-TKI-Tisle vs TACE-TKI	-0.87 (-1.50 , -0.25)	-0.89 (-1.50 , -0.25)	93.60%
	TACE-TKI-Tori vs TACE-TKI	-0.84 (-1.90 , 0.22)	-0.84 (-1.80 , 0.13)	-
	TKI-Camre vs TACE-TKI-Camre	1.20 (-0.08 , 2.40)	1.20 (0.01 , 2.30)	-
	TKI-Sinti vs TACE-TKI-Sinti	1.10 (-0.13 , 2.20)	1.10 (-0.05 , 2.20)	-
OS				
	TACE-Bev-Atez vs Bev-Atez	-0.54 (-1.10 , 0.05)	-0.54 (-1.10 , 0.04)	0.00%
	TKI-Camre vs HAIC-TKI-Camre	0.69 (0.13 , 1.20)	0.69 (0.14 , 1.20)	27.10%
	TACE-Bev-Atez vs TACE	-1.00 (-2.00 , 0.01)	-0.62 (-1.40 , 0.14)	44.60%
	TACE-Camre vs TACE	-0.91 (-1.80 , -0.06)	-0.94 (-1.70 , -0.18)	-
	TACE-TKI vs TACE	-0.43 (-1.30 , 0.44)	-0.25 (-0.64.0.15)	0.00%
	TACE-TKI-Camre vs TACE	-0.76 (-1.20 , -0.31)	-0.79 (-1.20 , -0.44)	0.00%
	TACE-TKI-Pembro vs TACE	-0.22 (-0.98 , 0.55)	-0.52 (-1.10 , 0.05)	72.10%
	TACE-TKI-Tisle vs TACE	-0.97 (-1.90 , -0.03)	-0.82 (-1.40 , -0.30)	0.00%

	TACE-TKI vs TACE-Bev-Atez	-0.09 (-1.20 , 0.99)	0.37 (-0.39 , 1.10)	44.10%
	TACE-TKI-Camre vs TACE-Camre	0.18 (-1.10 , 1.50)	0.15 (-0.64 , 0.91)	-
	TACE-TKI-Camre vs TACE-TKI	-0.55 (-0.92 , -0.24)	-0.54 (-0.86 , -0.26)	99.10%
	TACE-TKI-Pembro vs TACE-TKI	-0.58 (-1.40 , 0.20)	-0.26 (-0.84 , 0.30)	69.80%
	TACE-TKI-Sint vs TACE-TKI	-0.38 (-1.20 , 0.40)	-0.38 (-1.10 , 0.39)	-
	TACE-TKI-Tisle vs TACE-TKI	-0.54 (-1.00 , -0.09)	-0.56 (-1.00 , -0.15)	85.20%
	TACE-TKI-Tori vs TACE-TKI	-1.40 (-2.10 , -0.66)	-1.40 (-2.10 , -0.67)	0.00%
	TKI-Camre vs TACE-TKI-Camre	0.98 (0.25 , 1.70)	0.98 (0.26 , 1.70)	0.00%
	TKI-Sinti vs TACE-TKI-Sinti	0.59 (-0.25 , 1.40)	0.60 (-0.23 , 1.40)	-
ORR				
	TACE-Bev-Atez vs Bev-Atez	9.50 (2.30 , 68.00)	9.40 (2.3 , 76.00)	-
	TKI-Camre vs HAIC-TKI-Camre	0.73 (0.40 , 1.30)	0.73 (0.42 , 1.30)	86.10%
	TACE-Bev-Atez vs TACE	1.80 (0.72 , 4.50)	1.70 (0.93 , 3.30)	0.00%
	TACE-Camre vs TACE	1.30 (0.38 , 4.40)	1.30 (0.58 , 2.90)	-
	TACE-Durva vs TACE	1.40 (0.58 , 3.30)	1.40 (0.64 , 3.00)	-
	TACE-Bev-Durva vs TACE	1.50 (0.62. 3.50)	1.50 (0.69 , 3.20)	-
	TACE-TKI vs TACE	1.70 (0.39 , 7.70)	1.70 (1.10 , 2.50)	0.00%
	TACE-TKI-Camre vs TACE	2.10 (1.20 , 3.50)	1.90 (1.30 , 2.80)	64.20%
	TACE-TKI-Pembro vs TACE	1.70 (0.72 , 3.90)	2.10 (1.20 , 3.90)	71.70%
	TACE-TKI-Tisle vs TACE	2.70 (1.00 , 7.00)	2.80 (1.60 , 5.00)	0.00%
	TACE-TKI vs TACE-Bev-Atez	0.95 (0.39 , 2.30)	0.95 (0.50 , 1.80)	0.00%
	TACE-TKI-Camre vs TACE-Camre	1.40 (0.56 , 3.60)	1.40 (0.67 , 3.20)	-
	TACE-Bev-Durva vs TACE-Durva	1.10 (0.46 , 2.50)	1.10 (0.50 , 2.30)	-
	TACE-TKI-Camre vs TACE-TKI	1.10 (0.77 , 1.60)	1.10 (0.84 , 1.60)	97.00%
	TACE-TKI-Pembro vs TACE-TKI	1.70 (0.68 , 4.30)	1.30 (0.70 , 2.40)	49.10%
	TACE-TKI-Sint vs TACE-TKI	1.40 (0.52 , 3.90)	1.40 (0.55 , 3.70)	-
	TACE-TKI-Tisle vs TACE-TKI	1.70 (1.10 , 2.90)	1.70 (1.10 , 2.70)	62.20%
	TACE-TKI-Tori vs TACE-TKI	1.70 (0.90 , 3.20)	1.70 (0.95 , 3.10)	0.00%
	TKI-Camre vs TACE-TKI-Camre	0.40 (0.18 , 0.85)	0.40 (0.19 , 0.83)	0.00%
	TKI-Sinti vs TACE-TKI-Sinti	0.50 (0.18 , 1.40)	0.51 (0.18 , 1.30)	-
Safety				
	TACE-Camre vs TACE	1.00 (0.01 , 200.00)	1.00 (0.03 , 13.00)	-
	TACE-Durva vs TACE	1.20 (0.29 , 4.70)	1.20 (0.30 , 4.50)	-

TACE-Bev-Durva vs TACE	1.60 (0.38 , 6.10)	1.60 (0.40 , 6.00)	-
TACE-TKI-Camre vs TACE	2.10 (0.54 , 7.70)	2.00 (0.55 , 7.10)	0.00%
TACE-TKI-Pembro vs TACE	2.00 (0.43 , 9.60)	2.00 (0.44 , 9.40)	-
TACE-TKI-Camre vs TACE-Camre	1.8 (0.05 , 69.00)	1.9 (0.15 , 68.00)	-
TACE-Bev-Durva vs TACE-Durva	1.30 (0.33 , 5.20)	1.30 (0.34 , 5.20)	-
TACE-TKI-Camre vs TACE-TKI	2.20 (0.65 , 9.30)	2.00 (0.65 , 9.20)	43.70%
TACE-TKI-Tisle vs TACE-TKI	1.10 (0.42 , 3.20)	1.10 (0.42 , 3.20)	0.00%

OS=overall survival. PFS=progression-free survival. ORR=objective response rate. TACE=transcatheter arterial chemoembolization. HAIC=hepatic arterial infusion chemotherapy. TKI=tyrosine-kinase inhibitor. Bev=bevacizumab. Camre=camrelizumab. Pembro=pembrolizumab. Tisle=tislelizumab. Tori=toripalimab. Atez=atezolizumab. Sinti=sintilimab. Durva=Durvalumab.

Table.S8 Post-study treatment in follow-up

Study Name	Follow-up treatment	Arm	Patients who achieved downstaging (unique patients)			Number of patients with at least one treatment after disease downstaging, for maintaining the response of study treatment, or due to the intolerable toxicity								Number of patients with at least one treatment after disease progression								
			Surgery	LT	ablation	Surgery	ablation	RT	TACE or HAIC	MTAs	ICIs	MTAs plus ICIs	Combination therapy	surgery	ablation	RT	TACE or HAIC	MTAs	ICIs	MTAs plus ICIs	Chemo	Combination therapy
Zuo, 2024	yes	HAIC-TKI-Camre	54	-	-	124	56	45	47	486	369	214	-	11	15	10	17	42	20	4	-	-
		TKI-Camre	16	-	-	32	187	24	26	331	151	128	-	8	17	9	19	47	22	2	-	-
Li, 2024	unclear	HAIC-TKI-Camre	-	-	-	-	-	-	-	-	-	-	-	-	-	-	-	-	-	-	-	-
		TKI-Camre	-	-	-	-	-	-	-	-	-	-	-	-	-	-	-	-	-	-	-	-
Cao, 2023	unclear	TACE-Atez-Bev	-	-	-	-	-	-	-	-	-	-	-	-	-	-	-	-	-	-	-	-
		Atez-Bev	-	-	-	-	-	-	-	-	-	-	-	-	-	-	-	-	-	-	-	-
Zheng, 2024	yes	TACE-Atez-Bev	-	-	-	-	-	-	-	-	-	-	-	-	5	0	26	14	0	5	-	-
		TACE	-	-	-	-	-	-	-	-	-	-	-	-	16	4	67	38	0	12	-	-
Zhao, 2023	unclear	TACE-Atez-Bev	-	-	-	-	-	-	-	-	-	-	-	-	-	-	-	-	-	-	-	-
		TACE-TKI	-	-	-	-	-	-	-	-	-	-	-	-	-	-	-	-	-	-	-	-
Jin, 2023	unclear	TACE-TKI-Camre	-	-	-	-	-	-	-	-	-	-	-	-	-	-	-	-	-	-	-	-
		TACE	-	-	-	-	-	-	-	-	-	-	-	-	-	-	-	-	-	-	-	-
Tang, 2024	unclear	TACE-TKI-Camre	-	-	-	-	-	-	-	-	-	-	-	-	-	-	-	-	-	-	-	-
		TACE	-	-	-	-	-	-	-	-	-	-	-	-	-	-	-	-	-	-	-	-
Ju, 2021	unclear	TACE-TKI-Camre	-	-	-	-	-	-	-	-	-	-	-	-	-	-	-	-	-	-	-	-
		TKI-Camre	-	-	-	-	-	-	-	-	-	-	-	-	-	-	-	-	-	-	-	-
Guo, 2022	yes	TACE-TKI-Camre	-	-	-	-	-	-	-	9	-	4	-	-	5	-	-	-	-	-	-	-
		TKI-Camre	-	-	-	-	-	-	-	6	-	4	-	-	2	-	-	-	-	-	-	-
Chen, 2021	yes	TACE-TKI-Pembro	18	-	-	-	-	-	-	-	-	-	-	-	-	-	-	-	-	-	-	-
		TACE-TKI	8	-	-	-	-	-	-	-	-	-	-	-	-	-	-	-	-	-	-	-
Xiang, 2023	unclear	TACE-TKI-Camre	-	-	-	-	-	-	-	-	-	-	-	-	-	-	-	-	-	-	-	-
		TACE-TKI	-	-	-	-	-	-	-	-	-	-	-	-	-	-	-	-	-	-	-	-
Sun, 2022	unclear	TACE-TKI-Camre	-	-	-	-	-	-	-	-	-	-	-	-	-	-	-	-	-	-	-	-
		TACE-TKI	-	-	-	-	-	-	-	-	-	-	-	-	-	-	-	-	-	-	-	-
Lu, 2023	unclear	TACE-TKI-Tori	-	-	-	-	-	-	-	-	-	-	-	-	-	-	-	-	-	-	-	-

		TACE-TKI	-	-	-	-	-	-	-	-	-	-	-	-	-	-	-	-	-	-	-	-
Zhu, 2022	unclear	TACE-TKI-Camre	-	-	-	-	-	-	-	-	-	-	-	-	-	-	-	-	-	-	-	-
		TACE-TKI	-	-	-	-	-	-	-	-	-	-	-	-	-	-	-	-	-	-	-	-
Duan, 2023	unclear	TACE-TKI-Camre	-	-	-	-	-	-	-	-	-	-	-	-	-	-	-	-	-	-	-	-
		TACE-TKI	-	-	-	-	-	-	-	-	-	-	-	-	-	-	-	-	-	-	-	-
Liu, 2023 (1)	unclear	TACE-TKI-Camre	-	-	-	-	-	-	-	-	-	-	-	-	-	-	-	-	-	-	-	-
		TACE-TKI	-	-	-	-	-	-	-	-	-	-	-	-	-	-	-	-	-	-	-	-
Jiang, 2024 (1)	unclear	TACE-TKI-Camre	-	-	-	-	-	-	-	-	-	-	-	-	-	-	-	-	-	-	-	-
		TACE-TKI	-	-	-	-	-	-	-	-	-	-	-	-	-	-	-	-	-	-	-	-
Qu, 2022	yes	TACE-TKI-Tori	15	-	-	-	-	-	-	-	-	-	-	-	-	-	-	-	-	-	-	-
		TACE-TKI	4	-	-	-	-	-	-	-	-	-	-	-	-	-	-	-	-	-	-	-
Jiang, 2024 (2)	unclear	TACE-TKI-Tisle	-	-	-	-	-	-	-	-	-	-	-	-	-	-	-	-	-	-	-	-
		TACE-TKI	-	-	-	-	-	-	-	-	-	-	-	-	-	-	-	-	-	-	-	-
Zhao, 2024	unclear	TACE-TKI-Tisle	-	-	-	-	-	-	-	-	-	-	-	-	-	-	-	-	-	-	-	-
		TACE-TKI	-	-	-	-	-	-	-	-	-	-	-	-	-	-	-	-	-	-	-	-
Wu, 2025	unclear	TACE-TKI-Sinti	-	-	-	-	-	-	-	-	-	-	-	-	-	-	-	-	-	-	-	-
		TACE-TKI	-	-	-	-	-	-	-	-	-	-	-	-	-	-	-	-	-	-	-	-
Lang, 2023	unclear	TACE-TKI-Sinti	-	-	-	-	-	-	-	-	-	-	-	-	-	-	-	-	-	-	-	-
		TKI-Sinti	-	-	-	-	-	-	-	-	-	-	-	-	-	-	-	-	-	-	-	-
Chen, 2024 (1)	yes	TACE-TKI-Tisle	-	-	-	-	-	-	-	-	-	-	-	-	0	-	0	0	-	7	-	29
		TACE-TKI	-	-	-	-	-	-	-	-	-	-	-	-	0	-	0	3	-	2	-	24
		TACE	-	-	-	-	-	-	-	-	-	-	-	-	2	-	2	3	-	0	-	21
Wu, 2023 (1)	yes	TACE-TKI-Camre	-	-	-	-	-	-	-	-	-	-	-	-	-	1	3	14	0	5	-	3
		TACE-Camre	-	-	-	-	-	-	-	-	-	-	-	-	-	0	11	8	0	3	-	3
		TACE	-	-	-	-	-	-	-	-	-	-	-	-	-	0	3	14	2	5	-	3
Sangro,2025	yes	TACE-Durva-Bev	3	2	11	-	-	-	-	-	-	-	-	-	-	-	-	-	-	-	-	-
		TACE-Durva	3	4	21	-	-	-	-	-	-	-	-	-	-	-	-	-	-	-	-	-

		TACE	2	2	17	-	-	-	-	-	-	-	-	-	-	-	-	-	-	-	-	-
Kudo,2025	yes	TACE-TKI-Pembro	-	-	-	-	-	-	-	-	-	-	-	15	84	-	-	31	-	26	2	-
		TACE	-	-	-	-	-	-	-	-	-	-	-	12	104	-	-	43	-	47	2	-
Li,2025	unclear	TACE-Ate-Bev	-	-	-	-	-	-	-	-	-	-	-	-	-	-	-	-	-	-	-	-
		Ate-Bev	-	-	-	-	-	-	-	-	-	-	-	-	-	-	-	-	-	-	-	-
Cai, 2025	unclear	TAE-HAIC-Len-Tilse	-	-	-	-	-	-	-	-	-	-	-	-	-	-	-	-	-	-	-	-
		TAE-HAIC-Ate-Bev	-	-	-	-	-	-	-	-	-	-	-	-	-	-	-	-	-	-	-	-
Zhao, 2025	yes	HAIC-len-tisle	8	-	-	-	-	-	-	-	-	-	-	-	-	-	-	1	-	9	-	22
		len-tisle	1	-	-	-	-	-	-	-	-	-	-	-	-	-	-	4	-	10	-	41
Li, 2023 (1)	yes	TACE-TKI-Camre	-	-	-	-	-	-	-	-	-	-	-	-	-	-	-	8	-	16	-	9
Wu, 2023 (2)	yes	TACE-TKI-Camre	29	-	-	-	-	-	-	-	3	14	10	-	-	-	-	-	-	-	-	-
Li, 2023 (2)	yes	TACE-TKI-Camre	10	-	-	-	-	-	-	-	-	-	-	-	-	-	-	-	-	-	-	-
Cai, 2023	unclear	TACE-TKI-Sinti	-	-	-	-	-	-	-	-	-	-	-	-	-	-	-	-	-	-	-	-
Chen, 2024 (2)	yes	TACE-TKI-Enva	16	-	-	-	-	-	-	-	-	-	-	-	-	-	-	-	-	-	-	-
Zhang, 2023	yes	HAIC-TKI-Camre	5	-	1	-	-	-	-	-	-	-	-	-	-	-	-	-	-	-	1	-
He, 2023	yes	HAIC-TKI-Tori	1	-	-	-	-	-	-	-	-	-	-	1	1	2	10	-	-	-	1	-
Lai, 2022	yes	HAIC-TKI-Tori	8	-	-	-	-	-	-	-	-	-	-	-	-	-	-	-	-	-	-	-
Tai, 2021	yes	TARE-Nivo	-	-	-	-	-	-	-	-	-	-	-	1	-	1	1	20	4	2	4	-
Yu, 2024	unclear	TARE-Pembro	-	-	-	-	-	-	-	-	-	-	-	-	-	-	-	-	-	-	-	-
Liu, 2023 (2)	yes	HAIC-IBI305-Sinti	14	-	3	-	-	-	-	-	-	-	-	-	-	-	-	-	-	-	-	-
Ren, 2024	yes	TACE-TKI-Pembro-chemo	6	-	-	-	-	-	-	-	-	-	-	-	-	-	-	-	-	-	-	-
Mu, 2025	unclear	TACE-Sinti-Bev	-	-	-	-	-	-	-	-	-	-	-	-	-	-	-	-	-	-	-	-
Gao, 2025	yes	HAIC-TKI-Sinti	18	-	-	-	-	-	-	-	-	-	-	-	-	-	-	-	-	-	-	-
Shen, 2025	yes	HAIC-TKI-Camre	2	-	-	-	-	-	-	-	-	-	-	-	-	-	-	-	-	-	-	-
Chen,2023 (1)	yes	TAE-HAIC-TKI-Tisle	11	-	-	-	-	-	-	-	-	-	-	-	-	-	-	-	-	-	-	-

Chen,2023 (2)	yes	HAIC-TKI-Tisle	7	-	-	-	-	-	-	-	-	-	-	-	-	-	-	-	-	-	-	-

TKIs include lenvatinib, sorafenib, regorafenib, and apatinib.

MTAs plus ICIs include TKIs plus PD-1/PD-L1/CTLA-4 inhibitors and anti-VEGF antibodies plus PD-1/PD-L1 inhibitors.

Local ablation therapies include Radiofrequency ablation and Microwave ablation

Combination therapy denotes the integration of local treatment with systemic therapy in this context.

Abbrevations: TACE, transarterial chemoembolization; HAIC, hepatic artery infusion chemotherapy; TKIs, tyrosine kinase inhibitors; MTAs, molecular targeted agents; ICIs, Immune checkpoint inhibitors; PD-1, programmed death 1; PD-L1, programmed death-ligand 1; CTLA-4, cytotoxic T lymphocyte-associated antigen-4; VEGF, vascular endothelial growth factor; Chemo, Chemotherapy; RT, Radiotherapy, LT, Liver transplantation.
