# Supplementary material for: Comparative efficacy of combination regimens based on interventional therapy and immune checkpoint inhibitors (ICIs) in patients with intermediate- and advanced-stage hepatocellular carcinoma: a systematic review, meta-analysis, and network meta-analysis
Source: Cancer Immunol Immunother. 2026 Feb 7;75(3):67. doi: 10.1007/s00262-025-04251-5 (PMC12882918; doi:10.1007/s00262-025-04251-5)
Supplement: Supplementary file 2 — Supplementary file2 (DOCX 25026 kb) [file 262_2025_4251_MOESM2_ESM.docx]

**Supplementary figures**

**Figure S10 Flowchart of our study**

**
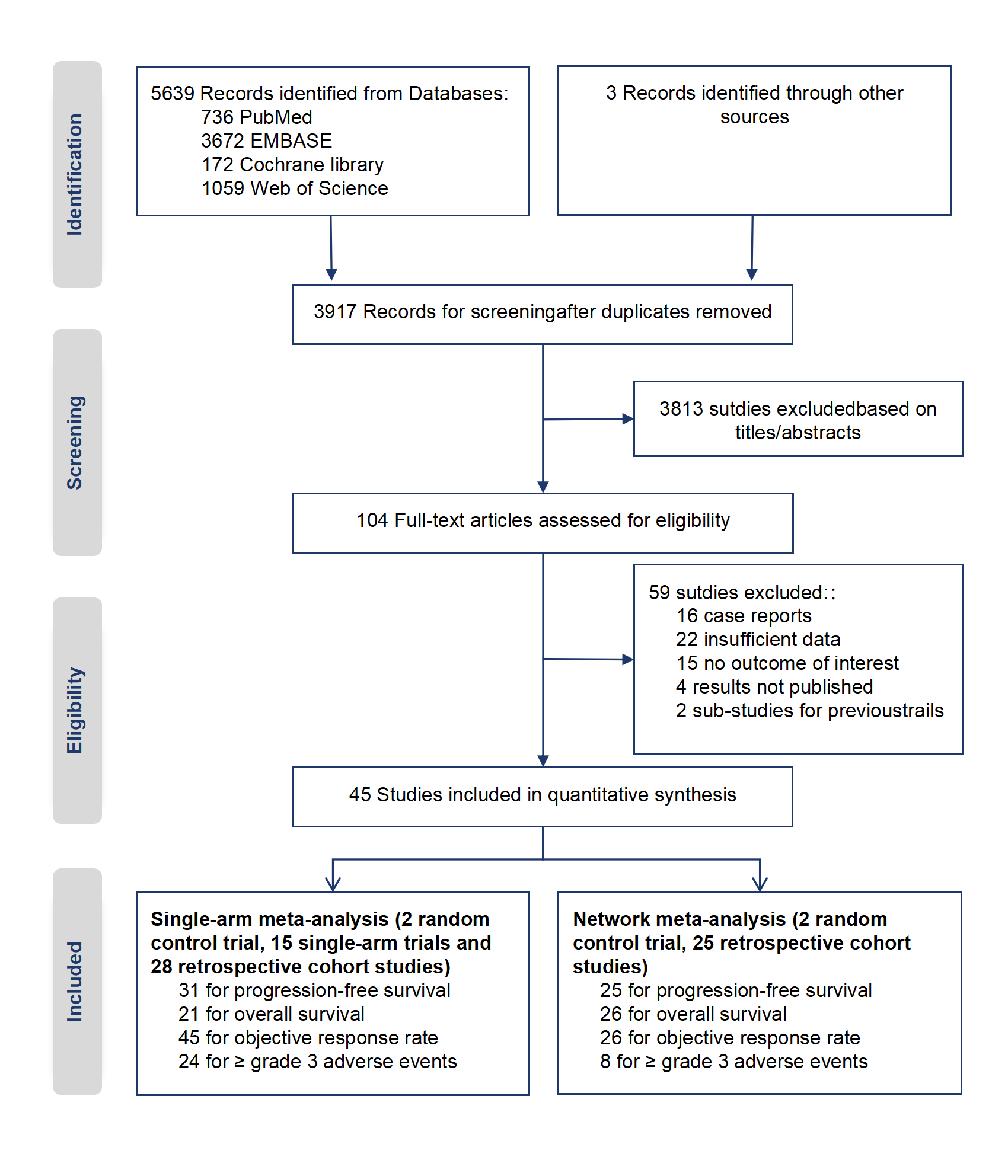
**

**Figure S11 Network diagrams of comparisons on different outcomes of treatments**


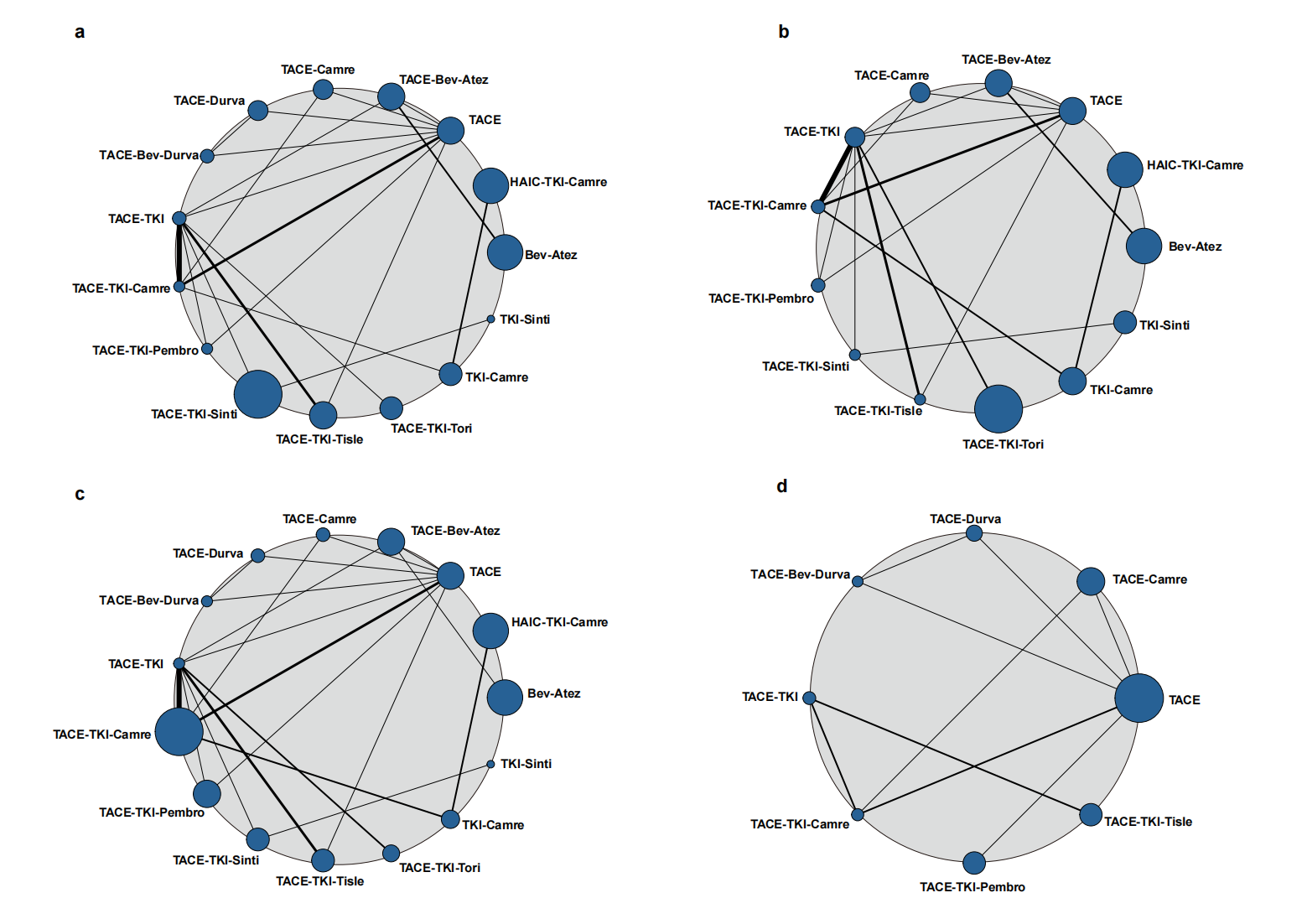


Comparisons were generated by using the Bayesian framework on (a) overall survival, (b) progression-free survival, (c) objective response rate, (d) grade ≥3 adverse events. Each circle represents an intervention as a node in the network. The size of the nodes reflects the number of patients treated, and the width of each edge is proportionally weighted according to the number of comparisons. TACE=transcatheter arterial chemoembolization. HAIC=hepatic arterial infusion chemotherapy. TKI=tyrosine-kinase inhibitor. Bev=bevacizumab. Camre=camrelizumab. Pembro=pembrolizumab. Tisle=tislelizumab. Tori=toripalimab. Atez=atezolizumab. Sinti=sintilimab. Durva=Durvalumab.

**Figure S12 The surface under the cumulative ranking curve (SUCRA) of network meta-analysis**


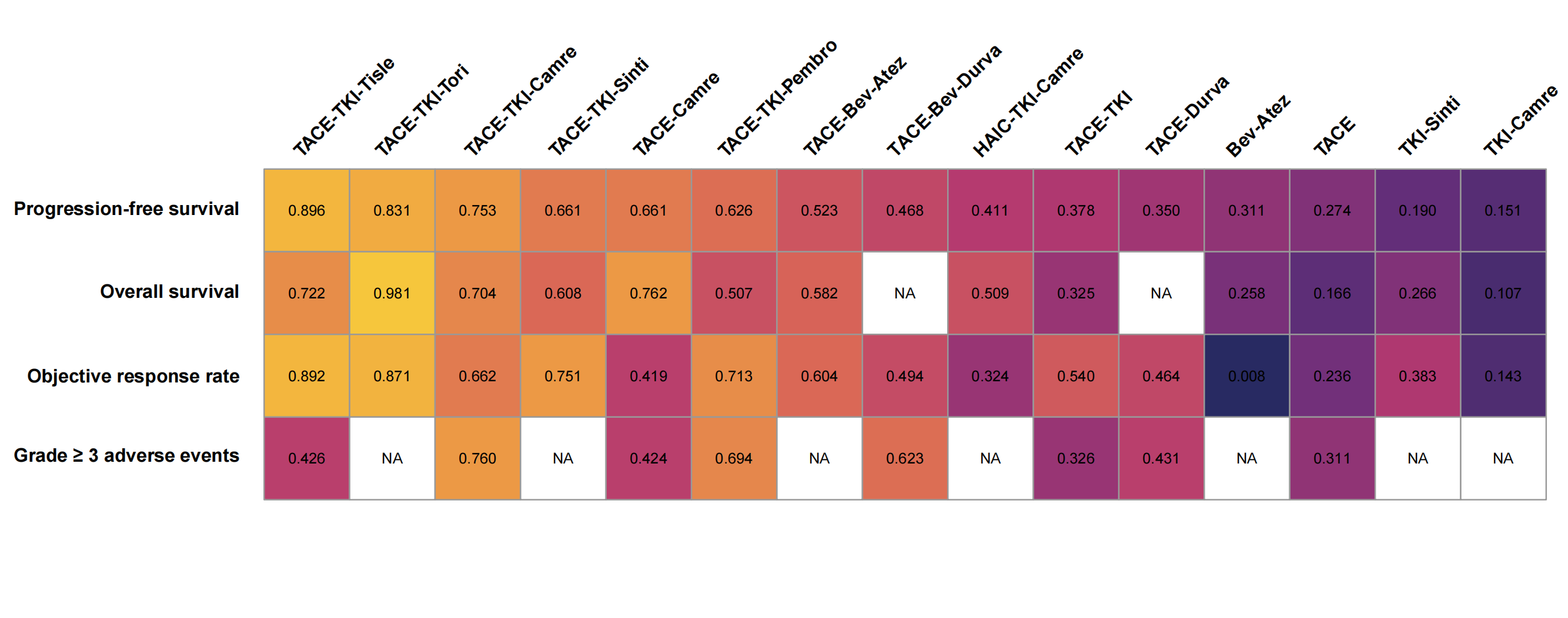


A SUCRA of 1 represents the best and 0 represents the worst. TACE=transcatheter arterial chemoembolization. HAIC=hepatic arterial infusion chemotherapy. TKI=tyrosine-kinase inhibitor. Bev=bevacizumab. Camre=camrelizumab. Pembro=pembrolizumab. Tisle=tislelizumab. Tori=toripalimab. Atez=atezolizumab. Sinti=sintilimab. Durva=Durvalumab.
